# Healthy adults’ views and experiences on behavior change strategies in mobile applications for diet monitoring: A single centre qualitative study

**DOI:** 10.1371/journal.pone.0292390

**Published:** 2023-11-16

**Authors:** Nur Melissa Abdul Khalil, Fadzilah Hanum Mohd Mydin, Foong Ming Moy

**Affiliations:** 1 Department of Primary Care Medicine, Faculty of Medicine, University of Malaya, Kuala Lumpur, Malaysia; 2 Department of Social & Preventive Medicine, Faculty of Medicine, University of Malaya, Kuala Lumpur, Malaysia; Swiss Paraplegic Research, SWITZERLAND

## Abstract

Mobile diet apps assist in tracking nutritional intake and managing healthy eating diets. Effective diet apps incorporate specific population-tailored behavior change strategies (BCS) for user engagement and adherence to healthy diets. Malaysians have their unique behaviors and customs surrounding food and diet. This study aims to explore the perceptions, views, and experiences of healthy Malaysian adults with diet monitoring apps, by focusing on the BCS that engages users to use diet mobile apps and adhere to a healthy diet. A qualitative approach utilizing semi-structured in-depth interviews was conducted using a topic guide based on the Theory of Planned Behavior and trigger materials. Twenty interviews were audio-recorded and transcribed verbatim. Five themes emerged from the data, which are; instilling self-awareness, closed online group support, shaping knowledge, personalization, and user-friendly design. Influence by one’s social circle and attractiveness of app features may initiate users’ interest and help them engage with mobile diet apps, but the app’s ability to raise awareness of progress and impart useful knowledge help them adhere and comply to a healthier diet in the long run. The results from this study may help improve the behavior change strategy features of mobile diet apps for Malaysian adults.

## Introduction

Diet applications (apps) are software applications available on mobile devices to track nutritional intake and manage diets to improve diet quality and health outcomes [1]. Types of diet apps are wide and varied, for example, calorie counting apps, including food diaries, meal planning apps, special diet apps, intermittent fasting, and apps providing food-based information [2, 3]. Worldwide, diet apps are very popular, as some apps have been downloaded as many as 50 million times [4]. However, a very limited number of apps for diet monitoring were created with the needs of healthy Malaysian adults in mind [5]. Most diet apps created for Malaysians tend to be for populations with specific needs or diseases such as cancer and renal disease [6, 7].

Malaysians’ healthy eating behaviors and food choices are influenced by their health consciousness, knowledge, and attitudes regarding healthy food, subjective norms, and perceived behavioral control [8]. Malaysia is an ethnically diverse nation where each race and ethnicity practices different customs and cultures. Cultural backgrounds make up part of subjective norms, which may influence the differences in food choices between individuals of different ethnicities in Malaysia [9]. An example of challenges in societal norms is the need for family members to be present at mealtimes, as the Malaysian food culture, in preparation and consumption, is very communal, especially during festivities [10, 11]. Other activities such as family, work, or social gatherings require participants to join in and eat together to avoid offense [10]. Malaysians place importance on social influences and less on sensory appeal when choosing the food they consume [9, 12]. These attitudes and expectations of Malaysians may cause a difference in preference for intervention in diet monitoring and should be considered an important aspect when designing an app for monitoring diet.

A study on the mobile health app use pattern conducted in Malaysia showed that participants noticed great benefits in health app use for tracking their health status, providing motivation, and helping them gain knowledge in health and fitness [13]. In addition, a study on healthy Malaysian adults’ acceptability and compliance with a mobile food diary application reported high levels of agreement with diet apps based on the apps’ perceived usefulness, ease of use, attitude, and perceived enjoyment towards using the mobile diet application [5]. On the contrary, a recent meta-analysis of health app use among Asian users reported that app use declines over time. However, only one out of the nine studies involved users with no known diseases (except for obesity) [14, 15]. This is consistent with the findings of another Malaysian study among adult patients, which found that patients were more inclined to use mobile health apps when the perceived usefulness, ease of use, and subjective norm were positive [16].

Technology is perceived as useful if it helps users perform their tasks better to achieve their goals [17]. In this case, the diet app helps users keep track of their dietary intake and improve diet quality and health outcomes [17]. The apps’ ease of use is also an important factor in diet app adoption and goes hand-in-hand with perceived usefulness [18, 19]. Diet apps that are easy to navigate contain an extensive food database, and provide access to the required tools and information while being accurate, are associated with higher retention rates [18, 20, 21]. On the other hand, a lack of awareness or knowledge of diet apps may pose a barrier to their adoption [22, 23]. Users may be aware of the existence of diet apps but are unaware of the specific functions or are not confident in knowing how to use these apps properly [21, 22]. App users also have difficulty incorporating diet apps into their daily routines [21]. Diet apps are more likely to be utilized if adapted to the user’s smartphone usage habits [20]. Internal dedication and motivation to use diet apps can both be a motivator and a barrier to using diet apps [21, 22]. Users find positive self-motivation from positive outcomes, which helps to continue app use [21]. Conversely, previous diet app users have reported that their motivation to use the diet app diminishes over time due to the high burden of data entry, diminishing interest, and motivation [22, 24].

Interventions used in diet monitoring, either face-to-face or online, come from different behavior change strategies (BCSs), which are observable and replicable components of behavior change interventions [25]. Efforts to change people’s habits and attitudes to prevent disease is another way to describe [26]. Thus, BCSs are methods used to achieve behavior change. Other terms used are behavior change techniques and behavior change mechanisms [27, 28]. These BCS were clearly described in a taxonomy by Abraham and Michie [27]. Human behavior, in specific contexts, may be explained and predicted using BCS, which are rooted in behavior change theories (BCTs) [29]. Designing behavior change interventions that yield preferable changes requires good understanding of BCTs, and applying them skillfully in research and practice [30]. An understanding of theory may guide intervention developers to measure more carefully and astutely to assess the impact of interventions [30]. BCTs can enhance interventions by identifying theoretical constructs that should be selected and by determining the fundamental BCS to be incorporated into the intervention [31]. Research has shown the Theory of Planned Behavior (TPB) to be one of the most implemented BCTs found in studies investigating diet monitoring intervention efficacy [29, 32, 33]. TPB suggests that intention influences behavior, which in turn is affected by a person’s attitude towards the behavior, the subjective norm (i.e., how those in their surrounding view the behavior), and their perceived control of the behavior [29].

Several reviews have attempted to determine the BCS in diet apps to various degrees [4, 33, 34]. Content analysis of diet apps has categorized them by the common BCSs used in the diet apps, such as providing feedback, goal-setting for healthy eating and cooking, decision-making for groceries or restaurants, self-monitoring of energy and nutrient intake, weight tracking, and planning social support and change [35, 36]. The method by which these BCSs are integrated into app features are varied, and some may even overlap. However, the BCS that tended to be missing from most health apps were time management, relapse prevention, agreeing on a behavioral contract, teaching users how to use prompts and cues, and prompt barrier identification [37]. In addition, most studies showed that incorporating evidence-based BCS features in commercial mobile apps for diet monitoring is generally low [36–41]. This calls into question the quality of commercial diet monitoring apps and the validity of apps’ claims regarding their effectiveness and safety, especially considering the popularity of such apps [39].

Currently, no studies are exploring the BCS features among healthy Malaysian adults for diet monitoring using mobile apps. Therefore, we aim to explore healthy Malaysian adults’ perceptions, views, and experiences on behavioral change strategies in mobile apps for diet monitoring to ascertain the most effective strategies to help improve diet quality, health outcomes, and adherence to a diet app.

## Materials and methods

### Study design

This is a qualitative study approach utilizing semi-structured interviews among the healthy adults in Malaysia.

### Ethics

The study protocol has been granted ethics approval by the University Research Ethics Committee (UM.TNC2/UMREC—824). All participants were given a participant information sheet and provided written consent before being interviewed for this study.

### Participants

Healthy Malaysian adults who had previously used or are currently using a mobile app for diet monitoring were recruited by a combination of purposive and convenience sampling to achieve maximum variance in sampling. Participants were recruited via emails and text messages to staff and students in a public tertiary education center. Recruitment and interviews occurred from July 2020 to February 2021. Eligible participants were 18 years old or older, owned a smartphone, had experience using a diet-related mobile app, and could communicate in Malay or English. Those diagnosed with chronic illness or who had any physical or mental disabilities were excluded from the study. Before being interviewed, all participants were given a Participant Information Sheet, which provided details on the research. The participants also completed an online questionnaire to provide demographic, lifestyle, and smartphone-related data. All participants provided informed consent to be interviewed.

### Procedure

We conducted semi-structured interviews using a topic guide based on the domains of the Theory of Planned Behavior (TPB). The first author, who acted as the interviewer, conducted the interviews, and is the only author who had access to information that could identify individual participants during and after data collection.

TPB was chosen because it is one of the most implemented behavior change theories in studies investigating diet monitoring intervention efficacy [29, 32, 33]. TPB suggests that behavior is influenced by intention, which in turn is affected by a person’s attitude towards the behavior, the subjective norm (i.e., how those in their surrounding view the behavior), and their perceived control of the behavior [29]. The TPB was created to change behavioral intentions by focusing on the domains of the person’s attitudes towards the behavior, subjective norms, and behavioral control, thus giving TPB an advantage for the application of changing behavior towards diet monitoring and weight management over other behavior change theories [40]. Apps based on theories are more effective than those without theoretical bases [31, 42, 43]. It is also important to use theory and specify BCS to inform intervention design [31, 42].

We have pilot-tested the topic guide, where an initial topic guide was created based on the ones used in previous studies and the TPB [18, 44]. The pilot topic guide was tested with four participants. Following the pilot interview, a discussion between researchers was held, and changes to the topic guide were determined to fulfill the study’s objectives. The following interviews were conducted using the improved topic guide in line with the study objectives, and the participants were also supplemented with trigger materials which helped illustrate the different types of BCSs that may be used in a diet app. Trigger materials assist participants in visualizing and providing their opinions. The topic guide and trigger materials can be found in the Supporting Information. All semi-structured interviews were recorded and transcribed verbatim in their respective languages. Face-to-face interviews were audio recorded and online interviews were video and audio recorded. Data were managed using QDA Miner Lite v1.4.1 software.

### Data analysis

The transcripts were then analyzed using the inductive thematic analysis method [45]. The 93 hierarchically clustered BCT taxonomy was used as a guide and starting point to create codes and categories during data analysis [46].

Researchers, including a clinician and a dietitian with a Masters and a Ph.D., with over ten years of research experience, and a postgraduate candidate involved in conducting this study [47]. No relationships with participants were formed prior to this study. All of the data was coded in English. Data from the interview conducted in the Malay language was directly coded in Malay by the researcher who had interviewed to maintain the original meaning of the interview. Any translation of final quotes was done by the researcher post-analysis and were double checked by the other researchers. All researchers involved in this study are fluent in both Malay and English. Initial coding of the first transcript was done by two researchers, where labels were attached to text segments that indicated important material concerning research questions. All three researchers then compared and iteratively revised the codes to develop a set of themes that captured the essence of the interviews. This set of codes and themes was used as a basis to analyze the subsequent transcriptions. The researchers compared the raw data with the emerging theme labels and definitions. The themes were further refined by merging, adding, and removing redundant themes. The six-step inductive analysis is repeated with each new transcription until no new themes emerge, where the data is considered saturated [48, 49]. The codes and themes resulting from the analysis were discussed between all three researchers to ensure the dependability of results. Precautions were taken to ensure confirmability of the data collected, by ensuring all researchers involved in this study are trained to perform qualitative studies, and they are aware to not let their own biases affect the interviews conducted [50].

### Trustworthiness

Careful consideration was taken into account to ensure the trustworthiness of the data during this study, based on the four criteria of credibility, transferability, dependability, and confirmability, to ensure rigor in research [51, 52]. Peer examination and debriefing were conducted via discussions between the three researchers during data analysis to ensure reflexivity and reduce researcher bias. The data were analyzed thematically, whereby the researchers reviewed all individual transcripts and coded them via an iterative process to find similarities across the experiences of the individual participants while maintaining their original meaning and ensuring the credibility of the data. Direct quotes from the participants were used as examples of the final themes in the report. The current study tested the transferability of previous studies, by using similar data collection methods, albeit applied to a different population [18–22, 44]. Transferability in this study was established by ensuring the population from which the participants of this study was obtained, had enough variability to represent the larger Malaysian population. The description of participants from this study was provided in the “Participants” section of the methods.

## Results

There were total of 20 interviews conducted. Seven interviews were carried out face-to-face, whereas the other 13 interviews were done online, due to lockdown during the COVID-19 pandemic. The interviews lasted an average of 83 minutes per interview. We noted data saturation was achieved at the 18^th^ participant. Data saturation is reached when the ability to obtain additional new information has been attained and when further coding is no longer feasible [48]. In the context of this study, data saturation occurs when no new themes pertaining to the views and experiences of using a diet monitoring app emerge from the interviews. The participants comprised 13 females and seven males, ranging from 20 to 45 years old. They consist of healthy Malaysian adults whose highest education level was a bachelor’s degree or higher, except for one participant whose highest education level was at secondary school. Participants’ socio-demographic details are provided in Table 1. As this was an explorative study, background details and data regarding the participants’ relationships with diet apps and how they view their own bodies, was collected prior to the interviews in hopes that it would provide some context. Regarding body image, the participants were simply asked if they considered themselves to be ‘underweight’, ‘about right’ or ‘overweight’. Participants were asked how active they considered their lifestyle to be, and were given the options to answer ‘sedentary’, ‘moderately active’, ‘very active’ or ‘athlete’. Participants were asked to rate their own diet, and they were given a 3-point Likert scale ranging from ‘not healthy’ to ‘healthy’. These responses are self-reported by the participants.

**Table 1:** Participants demographics.

Characteristic	Subcategory	Number
**Gender**	Female	13
	Male	7
**Ethnicity**	Malay	7
	Chinese	10
	Indian	2
	Others	1
**Age**	20–29	9
	30–39	9
	40–45	2
**Highest Education**	Secondary School	1
	Bachelor’s Degree	11
	Postgraduate Degree	8
**Type of Diet App Used**	Calorie tracking apps	17
	Intermittent fasting apps	2
	Both apps	1
**Smartphone Operating System**	Apple iPhone	7
	Android	13
**Average daily smartphones usage**	Less than 1 hour per day	6
	Less than 2 hours per day	6
	Less than 5 hours per day	4
	More than 5 hours per day	4
**Up to date with technology**	1 (Not up to date with technology)	0
	2	0
	3	10
	4	9
	5 (Very up-to-date with technology)	1
**Body Image**	Underweight	2
	About right	9
	Overweight	9
**Daily activity**	Sedentary	6
	Moderately active	10
	Very active	4
**Diet**	Not healthy	17
	Moderately healthy	2
	Healthy	1

Five themes emerged from the data; Instilling Self-Awareness, Closed Online Group Support, Shaping Knowledge, Personalisation, and User-Friendly Design, and the subthemes correlated with each themes are shown in Table 2.

**Table 2:** Subthemes and themes.

Subthemes	Themes
Awareness of Progress	**Instilling Self-Awareness**
Awareness of Consequences	
In-App Community	**Closed online group support**
Social Circle	
Accurate and Reliable Information	**Shaping Knowledge**
Personalized Goal Setting	**Personalization**
Personal Tailoring of Features	
Privacy Concerns	
Mental Stress	
Ease of Use	**User-Friendly Design**
Smooth User Experience	

### Instilling self-awareness

As diet apps are mainly used to track diets, and functions highlighting and displaying the users’ progress and the consequences help motivate the participants to comply with their targeted diet change. In addition, the users’ self-awareness of their progress and the consequences of not performing the target task helps motivate them to stay on track when developing their new dietary habits.

Participants tended to feel motivated to perform the target habits when they were *aware of the changes and progress* they were making. Although self-monitoring somewhat achieves this, having the progress be more apparent via providing tangible rewards, feedback, and encouragement on progress helps enhance users’ confidence and self-efficacy.

*“My goal is to know whether my diet currently is okay and whether I need to make any change to it… So from there, I generally have an idea of whether the food that I eat, what could be the long-term effect to me, yeah, I guess that’s the benefit of using an app.” (P5, Female, 24 years old)*

App features that allow for social comparison and identification of achievements, such as milestone badges and leaderboards, respectively, also assist in keeping participants aware of their progress.

*“One thing quite good is like, for example, regarding the food that you key in, they put in like, what is the content of the food, like there is an encouragement from it, like for example when you take like a lot of calcium, then it will say like "It’s a good amount," (P11, Female, 33 years old)*

*“I mean, for example, today’s intake, maybe you can put a smiley badge if you took fewer calories (than the calorie limit), an angry face if it’s over the calorie (limit).” (P13, Female, 31 years old)*

Using a diet app made the participants more *aware of the consequences* and outcomes of their behavior change. Seeing the record of their eating behaviors, along with feedback, or reminders provided by the app, made the participants realize the cause and effect of their eating behaviors, thus helping them make better food choices and meal plans.

*“It (My diet) does change. So, for example, when I noticed (my calorie intake is) less, then I will increase it, no matter if it’s the portion of the frequency or the type of food, for example, the method of cooking. So I realized that fried food contributes a lot of calories and fats, which I eat very little. So I will probably allow myself to take more of it when, like, occasionally.” (P5, Female, 24 years old)*

*“…This app also gives a graph (on my progress). If I ate like this every day so it will show me the graph for this week. (For example) on Wednesday I already exceeded the calorie goals, something like that. So I think it’s good for me, so it will make me realize, okay next week I must do better than this week.”(P8, Male, 27 years old)*

In contrast, some participants felt guilt or regret after seeing records of unhealthy eating behaviors, which may make them cease their use of the diet app. Some participants mentioned that they tend to ignore the feedback given.

*“… Because I have to track every meal that I took… So, sometimes when I key in and I notice I have been taking more (than my daily calorie limit). So… I don’t want to key in anymore… what I dislike is, because we need to keep… checking and keep keying in… and they send reminders as well if we did not key in. When I realise (calorie intake is more than target), I try to cut the necessary intake, but I was in denial, (I ignored it).” (P14, Female, 36 years old)*

Some participants find using the app and having to input data to be cumbersome and felt that reminders might help them.

*“I think I would like it (reminder to record meals) because I keep forgetting to key in, so I would like it like you know appear on my screen that says that I haven’t, yeah then I would key in. For me I like it.” (P11, Female, 33 years old)*

Overall, most participants agreed that using diet apps causes them to be more aware of what they eat, and how that affects their bodies. However, the response the participants have to this realisation differ, with some feeling more motivated to continue with the desired change, and others feeling demotivated and ceasing to use the diet app.

### Closed online group support

Participants appreciated continued social support, which helped participants adhere to app use and target behavior. Social support may be defined as social resources perceived to be available, or are provided by non-professionals, from both formal support groups or informal helping relationships [53]. In the case for users of mobile apps for diet monitoring, support may come from face-to-face contacts such as family and friends who use the same app or have the same healthy eating interests, or from online communities within mobile apps or social media. Our participants prefer more involved social support from an in-app community, but also would like to choose the people they interact with online.

*“I prefer in-app community rather than the other way (linking to other social media apps). Because for the in-app community I think one of the features that might be helpful would be to gather people of common goals together.”(P5, Female, 24 years old)*

*“It will be nice if the user can choose whether they want somebody they know or don’t know because some people might be concerned about sharing their food intake and their like stuff like BMI with people they know, they might feel it’s a related to body shaming and all that. So it’s always nice if the user can be in a healthy or friendly community while trying to achieve goals together, it’s more like a virtual support group.” (P5, Female, 24 years old)*

Support from family or real life friends are appreciated, but not necessarily utilized in depth (i.e. they may not specifically confide with them regarding challeges that they face in changing their lifestyle, especially if those family members do not have the same lifestyle goals). They value support from those who may have similar insight or experiences as them, and having personal connections with them may be regarded as a bonus.

*“Only with my friends, with my family I do talk about it, but they don’t really ask about what kind of apps I use.” (P15, Female, 22 years old)*

*“I think my my family, it’s more to a traditional mindset. So what is important to them is that you have to eat rice, and carbohydrates are not the evil thing… They don’t get it. So I don’t really talk about this to them, but I just do what I want.” (P10, Female, 20 years old)*

*“In a group effort you feel somebody supporting you, somebody doing the same thing, or somebody reminding each other, so like a closed group, like we all agreed to join this app, and we agree to eat, you know only this amount of food.”(P4, Female, 33 years old)*

### Shaping knowledge

Diet apps are also used as a source of diet information for the participants, thereby being central in *shaping participants’ knowledge* of eating habits and food choices. Diet apps may suggest meals and recipes as well as healthy food resources and provide instructions, such as health information.

*“They will put it on the main page, then they give you ideas on how to how to make healthy cooking. And also, how to maintain or what are things that we can do, to maintain fitness. Yeah, that’s what I like.” (P12, Female, 37 years old)*

However, the information shared in the app must be accurate and reliable. Mainly one participant specifically stated that the suggestions in the diet app should be vetted my a health professional.

*“I think it will be good to have some health information. Like food information, and health information, if you take too much of this lot (of food), it can lead to diseases. The relevant diseases. But not too lengthy.”(P4, Female, 33 years old)*

*“(The health articles and tips) give us some ideas (on what to do), but the content of what they write and put that should be vetted, maybe carefully selected by a nutritionist, or medical practitioners because we shouldn’t be giving people wrong ideas via write-ups and so on.” (P6, Female, 42 years old)*

### Personalization

The ability to modify app features per participants’ needs and preferences helps them engage with the app and provide confidence in reaching goals [54]. The *personalization* of a diet app may cover various aspects, namely goal setting and the personal tailoring of features for ease of use, and also fits participants’ privacy and mental health concerns.*Personalized Goal Setting* is usually one of the BCS features that participants encounter when using diet apps, as they will have to set a goal when creating their user profile. Participants feel that goal setting is important and effective for behavior change; however, they also feel that it needs to be coupled with other strategies, such as planning social support and personalized feedback, to be truly effective.

*“… I think the other goal (habit and behavior goals) is just secondary goals (to weight change). I think it’s good that they put it (wight goals and habit goals) together. When you know you want to lose some weight and then you know what kind of plan you want to proceed with.” (P8, Male, 27 years old)*

Having the app suggest suitable smaller goals that are personalized and realistic for the participant is also valued. For example, monthly or weekly weight goals may change as the app learns of the participants’ average daily calorie expenditure.

*“That time I control my food intake too, my goal is 1780 something kcal. So they give suggestions from the app… I think (the goals provided by the app) are possible to achieve…The apps put how many kilograms you want to lose in a week. So when you put more (kgs to lose), they advise you okay maybe you cannot achieve this because you don’t have a frequent exercise, maybe this is the best for you, so I chose the best for me…” (P8, Male, 27 years old)*

*Personal tailoring of features* is greatly appreciated by participants, as they liked having the diet app fit their needs, which may be achieved by the app providing personalized feedback, such as congratulating the user for reaching micronutrient targets or consuming the recommended about of fruit for the day. Participants appreciated the flexibility of choosing when and if they would like reminders to record their meals, as well as options to disable additional features. However, it must be clear to the user how they may set these features, which can be shown in a tutorial.

*“It’s always better to be able to customize it (reminders), provided that the tutorial is being given or the user is aware of the use, the function, the feature.” (P5, Female, 24 years old)*

*“I think warning (of going over daily calorie or nutrient limits) yes, I think the warning idea is, is really good. I think it’s really good but I think it’s better if you can enable it and disable it yourself, that would be much better.”(P1, Male, 25 years old)*

*“… One thing quite good is, for example regarding the food that you key in, they (the app) puts in the content of the food, then there is an encouragement from it, for example when you take like a lot of calcium, then it (the app) will say "It’s a good amount." (P11, Female, 33 years old)*

*Privacy concerns* related to how diet apps use and share their information. Participants are highly skeptical about the privacy and safety of their data and usually are not active on social media due to the same concerns. Most participants do not want a diet app tracking their whereabouts. Providing clear terms and conditions regarding the privacy and safety of their data and providing the user with an option to not link their account to any form of social media may help mitigate this concern.

*“I don’t allow it (location tracking). The reason is that I am scared the app will track where I am, it’s for safety reasons… I find the function interesting, but I am worried unwanted people will detect my whereabouts.”(P2, Female, 45 years old)*

*“I think you should have like a feature to either you enable it or you disable it (sharing on other social media) like if you really don’t want it then you just don’t post it la. I think that’s really important.” (P1, Male, 25 years old)*

*Mental stress* is also a reported concern from our participants as the feeling of restriction from diet and calorie tracking, which they feel may harm their mental health. Mainly two participants who experienced this either chose to take a break from using the app for a while before resuming or chose to shift to intermittent fasting apps. However, other participants also mentioned that they did not find mental health trackers in a diet app to be useful to them.

*"Only for a certain time, I don’t feel it’s good long term (strict diet tracking), it will mess up your mental health. Because you’d feel very restricted… I feel it’s okay to use it long term, but we have to take breaks in between, and not just focus on tracking (the diet pattern)." (P15, Female, 22 years old)*

*“I know it (mood tracker) will hardly (change my behavior), let’s say I eat ice cream because I’m stressed, I know it’s stress eating, that wouldn’t make a difference.” (P4, Female, 33 years old)*

### User-Friendly design

User-friendly design compromises three subthemes related to the appearance of the diet app, also known as user interface (UI). The UI is the part of a computer system with which the user interacts to perform their tasks and accomplish their goals [55]. Participants appreciated diet apps that are easy, smooth, and efficient to use whilst still being attractive and engaging. The *ease of app use* influences participants’ attitudes towards adopting a new habit of monitoring their diet and weight. Participants valued apps that are easy to navigate and understand whilst providing a wide range of relevant and accurate information. Overly complicated apps can be overwhelming, thus diminishing their interest. Free apps are also seen as accessible, but you may consider paying for a premium membership later on.

*“I think I just, because it’s simple and easy, and its user friendly as well. So, I don’t need to look for any guide, I can just open and fill up whatever question they ask.”(P14, Female, 36 years old)*

*“… I think that app is not by Malaysian, so the food that they put in is like very hard to put specific, like what kind of foods that we eat… not correlate to the calories. So I find it very difficult to use the apps…”(P11, Female, 33 years old)*

Free apps are also seen as accessible, but they may consider paying for a premium membership later on. Most participants preferred free apps, with it being on of their crirteria when selecting a diet app to try out.

*“Downloading is free but after that, there’s a kind of membership you can purchase. So there’s like a minimal amount, a few Ringgits only. Two or three Ringgits or what for a short duration. So I just wanted to test what functions they give.”(P6, Female, 42 years old)*

*“Yeah, if it’s paid app, then I think I think it will refrain people from using it, because there’s so many free apps, then why do they need to pay you?” (P12, Female, 37 years old)*

*Smooth user experience* (UX) is the experience created and shaped through technology and relates to impressions of the app after the user has passed the initial introduction stage [56]. Our participants have expressed interest in recording meals via taking pictures of their meals, as it is convenient, but have shown concerns over the pictures taking up phone memory storage and leading to glitches that slow down the app. Advertisements and unwanted pop-ups also take up time, which they find bothersome. Flexibility is valued in cases where the participant is unable to record their meals when they are busy; they may do so at another time. Participants were willing to explore the apps on their own rather than following tutorial guidance, as they felt following a tutorial is time-consuming. Concerning time, when the user finds the app to be tedious to use, it hinders them from continuing app use. This also happens when the user is unable to find suitable information when recording their meals. Thus, an attractive user interface, including eye-catching graphics and an easy-to-navigate layout, is important.

*“I mean like when I was too busy in the day, but I just remember when the last time I start my fast and when I end, so I just remember so I just key in (later).” (P13, Female, 31 years old)*

Rewards, such as vouchers and badges with encouraging quotes are valued. Regular updates to app features and provision of unlocking new app features and rewards are also appreciated, as it maintains an exciting sense of novelty to the app. However, they must be realistic and attainable.

*"I will like to look at the badges (with encouraging quotes), I mean like target completed sounds so encouraging." (P11, Female, 33 years old)*

*“I like the unlocking part (of badges and rewards). Because it actually gives you a bit of satisfaction, when you unlock something. But it also a cons because when you want to choose another level, you cannot proceed to it because it’s not unlocked.” (P10, Female, 20 years old)*

## Discussion

Our study found that Malaysian healthy adults’ perceptions, views, and experiences on behavioral change strategies in mobile apps for diet monitoring is consistent with those of previous studies, with differences in emphasis on certain features and societal norms regarding diet apps and diet change [5, 6]. There are five themes on behavioral changes strategies that emerged from this study, which are, instilling self-awareness, closed online group support, shaping knowledge, personalisation, and user-friendly design.

Malaysian healthy adults prefers apps that instill awareness of one’s current diet and include features that support, and foster behavior change help engage users and encourage them to comply with healthier diets, with differences in emphasis on quick-capture features and societal norms regarding mobile apps for diet monitoring [5, 6]. The increased knowledge and awareness of diet were generally agreed by the Malaysian healthy adults to aid in choosing healthier foods for future meals, leading to increased perceived behavioral control, which mirrors results from a study conducted in Norway [44]. However, they felt guilt when they were being updated on the progress and the consequences of their eating behavior, which can either be perceived negatively, causing them to stop using the app, or spur their motivation to improve their diet in the future. Features such as reminders via app notification, may assist with bringing awareness to user, and are are one of the main reasons Malaysian adults choose to use a mobile app for diet monitoring. The present study confirms the findings that prompt from mobile apps for diet monitoring are useful, but users prefer to be in control of them, as Malaysian adults find app notifications when they are busy to be bothersome [20].

In the context of our study, personalization refers to features that allow the user to tailor the app to their needs, allowing them to enable features that interest them, which may help the user engage with the app better [57]. One of the aspects highlighted by Malaysian mobile app for diet monitoring users was goal setting and how they felt having personalized goal setting was important to them. However, they did recognize that personalized goal setting still needs to be accompanied by other BCSs to effectively change their eating behavior [58]. This is in line with the themes of personalized guidance in a previous study, where the participants appreciated a more personalized coaching and guidance aspect of mobile apps for diet monitoring [22]. Malaysian adults preferred to have the liberty to tailor the apps to their needs, as it changes over time and differ person to person. For example, some Malaysian app users like having reminders and warnings in a mobile app for diet monitoring, while others do not like it if a reminder or warning were to pop up when they are busy. Being able to choose which feature is enabled in the mobile app for diet monitoring during setup will fix this issue. This concurs with prior studies, where the participants also highlighted that they did not like feeling that their lives were dictated by an app [20, 44].

In line with past studies, healthy Malaysian adults have expressed concern about who has access and control over the data they provide to mobile apps for diet monitoring [20, 21, 44]. This is consistent with the literature that suggests mobile app for diet monitoring users are hesitant to share information due to wanting to keep their information private [19, 21]. Allowing the user to personalize who has access to their data may put those who are sceptical about mobile apps for diet monitoring at ease, for example allowing the user to choose whether they would like to share their information with social media. However, when it comes to closed online group support, interestingly some Malaysian adults have opposing views compared to a past study [20]. Malaysian adults preferred to share their progress and diet information with like-minded individuals via an in-app community instead of other social media platforms, whereas a past study from the United Kingdom showed participants being averse to include online social contacts in their diet and behavior change [20]. This may be due cultural differences in social media acceptance, or the collectivistic culture in Malaysia, and may also be the reason why healthy Malaysian adults feel they are more likely to be able to adhere to using a mobile app for diet monitoring and a healthier diet when they feel supported by friends and family, who join them in the lifestyle change and diet or by giving positive encouragement, which mirrors past studies in other countries [18, 20, 44]. This relates to subjective norms in the TPB, which is described as the perceived social pressure to perform or not perform the behavior [29].

Similar to previous studies, it is apparent that Malaysian adults appreciated having a wide range of information on the go [20, 22]. However, there is a great emphasis on reliable nutrition information that is tailored to Malaysian cuisine, which tends to be missing in many commercial mobile app for diet monitorings. This is particularly important as nutrition knowledge greatly influences a person’s food intake decisions and eating habits [59]. An app built with this in mind would have a great advantage over foreign-built apps in the Malaysian market. Malaysian adults have also expressed concern that information shared in the app should be from reliable and credible sources, which is consistent with a previous study [20]. A possible explanation for this may be that commercial apps tend to crowdsource their data, which may cause some inaccuracies. A limitation that Malaysian apps may currently face is that the latest Malaysian Food Composition Database was created in the year 1997, and many new foods that have come into the market over recent years are not reflected in the database, although efforts are currently in place to update the information [60].

The findings from this study indicate that Malaysian adult mobile app for diet monitoring users appreciate a user-friendly app design with a simple and attractive layout whilst being easy to navigate and intuitive to use. This is in agreement with a study among adult users who felt those apps that are not straightforward to set up and use are undesirable [18]. Malaysian adults shared similar sentiments regarding pop-ups and advertisements, as they found them irksome. Previous studies showed mobile app for diet monitoring users were concerned that data collected in apps are used to target advertisements to them [20, 22]. Malaysian adults preferred regularly updated apps that remain relevant to the current times, but frequent updates were deemed to be bothersome. This finding to our knowledge, was not highlighted in previous studies. Overall, a simple app design and minimizing unnecessary features may help in achieving this.

Our findings have also highlighted that Malaysian adults are more likely to start using a mobile app for diet monitoring when it is free to download. Free apps are perceived as more accessible than paid apps by participants. Similar outcomes were reported by a study on mobile apps to facilitate self-care [19]. However, the previous study included apps for the management of chronic conditions; thus, it was unable to ascertain if results would differ between healthy and ill app users. Prior work identified that mobile app for diet monitoring users downloaded free apps due to their disposability which differs from our study, whereby participants downloaded free apps for their accessibility [20]. This difference may be due to most apps being developed overseas; thus, the payment for the apps is not in Malaysian Ringgit and is seen as expensive to our participants. However, our participants expressed openness to a premium membership at a low cost for a short duration.

### Strengths and limitations

Ensuring the dependability of results is one of the hallmarks of a good explorative qualitative study, and this was achieved by the detailed descriptions of the methods used in this study, from the purpose of performing this study, which was explained by the gaps in knowledge of the present literature, and the decision trail described in the analysis of the data collected [50]. The codes and themes resulting from the analysis were also discussed between the two researchers to ensure the dependability of the results. Precautions were taken to ensure the confirmability of the data collected were taken by ensuring both researchers involved in this study were trained to perform qualitative studies, and they were aware to not let their own biases affect the interviews conducted [50]. The topic guide and trigger materials used during the interview prompted the participants to talk about the main topic at hand, and the interviewers followed the participants’ direction in the conversation rather than leading them. Member checking was also performed, where the participants were contacted after the interview to confirm the interview transcript, to ensure the credibility of the data. However, only twelve participants responded. The researchers had also shared the final themes with the participants, and those who responded agreed with the themes.

The strength of the present study includes combining the use of TPB and the 93-item taxonomy to guide data collection and analysis to provide a well-rounded exploration of the perceptions, views, and experiences of healthy Malaysian adults with mobile apps for diet monitoring [27, 29]. Our study has managed to capture a wide variety of insights, including those who had positive and negative experiences, as well as those who felt indifferent about the apps they used.

The participants of this study are from diverse backgrounds of ethnicities and education levels; however, the limitations of this study should be noted. The participants were selected from a small demographic area within a university, with all of them being comfortable and relatively up-to-date with technology, and most of them being highly educated. Although the sample taken was sufficient for this exploratory study, further research, which includes participants of a lower socioeconomic status and those less competent with technology or with differing educational backgrounds, may be informative.

Duration of last app usage was not included as a criteria for selection, as we would like to collect a wide views from participants who had positive and negative experiences from using mobile diet apps. However we note that in chosing this criteria, it may create a limit to the study. We would suggest adding this criteria in future studies.

Applications of the findings of this study to populations outside of Malaysia is unknown, as it would depend on the population it is being applied to. However, similarities of the findings of this study, with the findings from similar past studies should be noted, as it also may predict findings for studies in other populations [18–22, 44]. Nevertheless, as past studies were carried out in Western populations, the findings from this study may provide more accurate depictions of results that may be obtained within Asian populations and societies that may hold similar values and lifestyles as Malaysia.

We have elected to utilize IDI in this study; nevertheless, the data obtained in this study is sufficient in answering the research question of the perceptions, views, and experiences of healthy Malaysian adults on the BCS used in mobile apps for diet monitoring.

### Implication for practice and research

This study has uncovered the main concepts and features that healthy Malaysian adults value in diet apps. Ensuring the app has a simple but distinctive interface and contains reliable nutrition and diet information that is catered to Malaysian ingredients and cuisine would set the app apart from other apps in the market. An app that allows users to interact with each other but also allows them the flexibility to opt out of certain features would be ideal.

These findings may be useful for integrating evidence-based BCS features into a mobile app for diet monitoring for healthy Malaysian adults. The following explains in further detail the types of app features or aspects of design that we would recommend based on each theme found in this study, along with the correlating BCS as listed by Abraham and Michie’s taxonomy [27]. Based on the definitions of the BCS provided by Abraham and Michie in their taxonomy, the themes of instilling self-awareness comprises of the BCSs of “providing information about behavioral health link,” “providing information on consequences,” and “providing feedback on performance” [27]. The features that correlate with these BCSs include summaries of performance that may be shown daily, weekly or monthly, and health tips and articles on the dangers or benefits of consuming certain foods. Closed online group support in diet apps helps users by providing information on others’ approval and also opportunities for social comparison [27]. These may be included in diet apps in the form of leaderboards, forums, and links to share the apps with friends. A comprehensive list of suggestions on how the BCS features may be applied to a diet app can be found in Table 3.

**Table 3:** Suggestion of BCS features.

No.	Themes	Behavior Change Strategy	App Feature/ Aspect of Design
1.	Instilling Self-Awareness	• Provide information about behavioral health links. • Provide information on consequences. • Provide feedback on performance	• Daily/weekly/monthly summaries of performance • Health tips or articles on the dangers or benefits of consuming too much or too little of certain foods.
2.	Closed Online Group Support	• Provide information about others’ approval • Provide opportunities for social comparison	• Leaderboard • Forums • Link to share the diet app with friends.
3.	Shaping Knowledge	• Provide instruction	• Recipes and articles on how to prepare healthy meals. • Health tips on how to incorporate more of certain food into one’s diet.
4.	Personalization	• Prompt specific goal setting • Set graded tasks • Prompt practice	• Provide suggestions for goals and tasks which users may choose (e.g., A range of weight that users may safely lose or gain in one month). • Suggest new goals based on users’ progress (e.g., milestones and badges) • Allowing users to enable or disable reminders, notifications, and certain features (i.e., Privacy settings, mental health reminders).
5.	User-Friendly Design	*This theme does not correlate with any BCS in particular but is still an important aspect of design to engage with diet app users.	• Simple but attractive colorway. • Clear illustrations (e.g., Graphs and diagrams) which are easy to understand.

Further quantitative research with a larger cohort may help confirm the findings of this study. The results of this study may be used as a basis for creating a questionnaire for future quantitative studies. We would suggest screening participants for eating disorders, as well as exploring communities with different socioeconomic backgrounds for future studies.

## Conclusion

This study has uncovered the main concepts and features which healthy Malaysian adults valued in diet apps. The vast perceptions of healthy Malaysian adults demonstrate that user-friendly diet apps, with attractive and straightforward features, motivate users to use the app, whereas social influences and new knowledge provided by the app help users to continue using the app for behavior change. The revelation of these concepts may be used to inform the design of apps for diet monitoring catered to Malaysian adults in the future.

## Supporting information

S1 File (DOCX)

S2 File (PDF)

S3 File (PDF)

S4 File (PDF)

S5 File (PDF)

## References

[1] Margaret Alic P. Diet Apps 2018 [Available from: https://reference.jrank.org/diets/Diet_Apps.html#:~:text=Diet%20apps%20are%20software%20applications,blood%20pressure%2C%20and%20heart%20disease.

[2] Flaherty S-J, McCarthy M, Collins A, McAuliffe F. Can existing mobile apps support healthier food purchasing behaviour? Content analysis of nutrition content, behaviour change theory and user quality integration. Public Health Nutrition. 2018;21(2):288–98. doi: 10.1017/S1368980017002889 29081322 PMC10261053

[3] Parmar A. A Detailed Guide on Developing a Diet and Nutrition Tracking App prismetric.com: Prismetric; 2022 [updated 11 Oct, 2022. Available from: https://www.prismetric.com/diet-and-nutrition-tracking-app-development-cost/.

[4] Ferrara G, Kim J, Lin S, Hua J, Seto E. A Focused Review of Smartphone Diet-Tracking Apps: Usability, Functionality, Coherence With Behavior Change Theory, and Comparative Validity of Nutrient Intake and Energy Estimates. JMIR Mhealth Uhealth. 2019;7(5):e9232. doi: 10.2196/mhealth.9232 31102369 PMC6543803

[5] Chen YS, Wong JE, Ayob AF, Othman NE, Poh BK. Can Malaysian young adults report dietary intake using a food diary mobile application? A pilot study on acceptability and compliance. Nutrients. 2017;9(1):62. doi: 10.3390/nu9010062 28098770 PMC5295106

[6] Ho CY, Ban ZH, Ng WH, Neoh MK, Jamhuri N, Abd Rahman Z. Feasibility study of smartphone application for self-monitoring dietary intake among cancer patients. Journal of Medical Research and Innovation. 2020;4(2):e000209–e.

[7] Lim JH, May Lim CK, Ibrahim I, Shahrul J, Mohd Zabil MH, Zakaria NF, et al. Development of a Personalized Apps to Inculcate Dietary Self-Management for Maintenance Hemodialysis Patients: A Need Analysis. Current Developments in Nutrition. 2020;4.

[8] Mamun AA, Hayat N, Zainol NRB. Healthy eating determinants: A study among Malaysian young adults. Foods. 2020;9(8):974. doi: 10.3390/foods9080974 32717851 PMC7466180

[9] Prescott J, Young O, O’neill L, Yau N, Stevens R. Motives for food choice: a comparison of consumers from Japan, Taiwan, Malaysia and New Zealand. Food quality and preference. 2002;13(7–8):489–95.

[10] Johari MZ. Strategies and factors influencing weight management in Malaysia. Newcastle University: Newcastle University; 2019.

[11] Raji MNA, Ab Karim S, Ishak FAC, Arshad MM. Past and present practices of the Malay food heritage and culture in Malaysia. Journal of Ethnic Foods. 2017;4(4):221–31.

[12] Othman KI, Ab Karim MS, Karim R, Adzhan N, Halim NA, Osman S. Factors influencing fruits and vegetables consumption behaviour among adults in Malaysia. Journal of Agribusiness Marketing. 2012;5:29–46.

[13] KC B, Alrasheedy A, Goh BH, Blebil A, Bangash NSA, Mohamed Ibrahim MI, et al. The Types and Pattern of Use of Mobile Health Applications Among the General Population: A Cross-Sectional Study from Selangor, Malaysia. Patient Preference and Adherence. 2021;Volume 15:1755–62. doi: 10.2147/PPA.S325851 34408408 PMC8367216

[14] Ang SM, Chen J, Liew JH, Johal J, Dan YY, Allman-Farinelli M, et al. Efficacy of Interventions That Incorporate Mobile Apps in Facilitating Weight Loss and Health Behavior Change in the Asian Population: Systematic Review and Meta-analysis. Journal of Medical Internet Research. 2021;23(11):e28185. doi: 10.2196/28185 34783674 PMC8663646

[15] Tanaka K, Sasai H, Wakaba K, Murakami S, Ueda M, Yamagata F, et al. Professional dietary coaching within a group chat using a smartphone application for weight loss: a randomized controlled trial. Journal of Multidisciplinary Healthcare. 2018;Volume 11:339–47. doi: 10.2147/JMDH.S165422 30038502 PMC6052929

[16] Yee TS, Seong LC, Chin WS. Patient’s intention to use mobile health app. Journal of Management Research. 2019;11(3):18–35.

[17] Davis FD. Perceived usefulness, perceived ease of use, and user acceptance of information technology. MIS quarterly. 1989:319–40.

[18] Tang J, Abraham C, Stamp E, Greaves C. How can weight‐loss app designers’ best engage and support users? A qualitative investigation. British journal of health psychology. 2015;20(1):151–71. doi: 10.1111/bjhp.12114 25130682

[19] Anderson K, Burford O, Emmerton L. Mobile health apps to facilitate self-care: a qualitative study of user experiences. PLoS One. 2016;11(5):e0156164. doi: 10.1371/journal.pone.0156164 27214203 PMC4876999

[20] Dennison L, Morrison L, Conway G, Yardley L. Opportunities and challenges for smartphone applications in supporting health behavior change: qualitative study. Journal of medical Internet research. 2013;15(4):e86. doi: 10.2196/jmir.2583 23598614 PMC3636318

[21] Lieffers JR, Arocha JF, Grindrod K, Hanning RM. Experiences and perceptions of adults accessing publicly available nutrition behavior-change mobile apps for weight management. Journal of the Academy of Nutrition and Dietetics. 2018;118(2):229–39. e3. doi: 10.1016/j.jand.2017.04.015 28625662

[22] Peng W, Kanthawala S, Yuan S, Hussain SA. A qualitative study of user perceptions of mobile health apps. BMC public health. 2016;16(1):1–11. doi: 10.1186/s12889-016-3808-0 27842533 PMC5109835

[23] Haithcox-Dennis M, Brinkley J, Richman A, DeWeese A, Byrd JL III. Mhealth on campus: assessing undergraduates attitudes and utilization of mobile health applications. Global Journal of Health Education and Promotion. 2012;15(1).

[24] Krebs P, Duncan DT. Health App Use Among US Mobile Phone Owners: A National Survey. JMIR mHealth and uHealth. 2015;3(4):e101. doi: 10.2196/mhealth.4924 26537656 PMC4704953

[25] Morrissey EC, Corbett TK, Walsh JC, Molloy GJ. Behavior change techniques in apps for medication adherence: a content analysis. Am J Prev Med. 2016;50(5):e143–e6. doi: 10.1016/j.amepre.2015.09.034 26597504

[26] WHO WHO. The world health report 2002: reducing risks, promoting healthy life: World Health Organization; 2002. 10.1080/1357628031000116808 14741909

[27] Abraham C, Michie S. A taxonomy of behavior change techniques used in interventions. Health psychology. 2008;27(3):379. doi: 10.1037/0278-6133.27.3.379 18624603

[28] West JH, Belvedere LM, Andreasen R, Frandsen C, Hall PC, Crookston BT. Controlling Your “App”etite: How Diet and Nutrition-Related Mobile Apps Lead to Behavior Change. JMIR Mhealth Uhealth. 2017;5(7):e95. doi: 10.2196/mhealth.7410 28694241 PMC5525004

[29] Ajzen I. The theory of planned behavior. Organizational behavior and human decision processes. 1991;50(2):179–211.

[30] Grol RPTM Bosch MC, Hulscher MEJL Eccles MP, Wensing M. Planning and Studying Improvement in Patient Care: The Use of Theoretical Perspectives. Milbank Quarterly. 2007;85(1):93–138. doi: 10.1111/j.1468-0009.2007.00478.x 17319808 PMC2690312

[31] Webb T, Joseph J, Yardley L, Michie S. Using the internet to promote health behavior change: a systematic review and meta-analysis of the impact of theoretical basis, use of behavior change techniques, and mode of delivery on efficacy. Journal of medical Internet research. 2010;12(1):e4. doi: 10.2196/jmir.1376 20164043 PMC2836773

[32] Tang J, Abraham C, Greaves C, Yates T. Self-directed interventions to promote weight loss: a systematic review of reviews. Journal of medical Internet research. 2014;16(2):e58. doi: 10.2196/jmir.2857 24554464 PMC3961624

[33] Zhao J, Freeman B, Li M. Can mobile phone apps influence people’s health behavior change? An evidence review. Journal of medical Internet research. 2016;18(11):e287. doi: 10.2196/jmir.5692 27806926 PMC5295827

[34] Greaves CJ, Sheppard KE, Abraham C, Hardeman W, Roden M, Evans PH, et al. Systematic review of reviews of intervention components associated with increased effectiveness in dietary and physical activity interventions. BMC Public Health. 2011;11(1):119. doi: 10.1186/1471-2458-11-119 21333011 PMC3048531

[35] Mauch CE, Wycherley TP, Laws RA, Johnson BJ, Bell LK, Golley RK. Mobile Apps to Support Healthy Family Food Provision: Systematic Assessment of Popular, Commercially Available Apps. JMIR mHealth and uHealth. 2018;6(12):e11867. doi: 10.2196/11867 30578213 PMC6320405

[36] Azar KM, Lesser LI, Laing BY, Stephens J, Aurora MS, Burke LE, et al. Mobile applications for weight management: theory-based content analysis. Am J Prev Med. 2013;45(5):583–9. doi: 10.1016/j.amepre.2013.07.005 24139771

[37] Direito A, Pfaeffli Dale L, Shields E, Dobson R, Whittaker R, Maddison R. Do physical activity and dietary smartphone applications incorporate evidence-based behaviour change techniques? BMC Public Health. 2014;14(1):646. doi: 10.1186/1471-2458-14-646 24965805 PMC4080693

[38] Chen J, Cade JE, Allman-Farinelli M. The Most Popular Smartphone Apps for Weight Loss: A Quality Assessment. JMIR mHealth uHealth. 2015;3(4):e104. doi: 10.2196/mhealth.4334 26678569 PMC4704947

[39] Rivera J, McPherson A, Hamilton J, Birken C, Coons M, Iyer S, et al. Mobile Apps for Weight Management: A Scoping Review. JMIR mHealth and uHealth. 2016;4(3):e87. doi: 10.2196/mhealth.5115 27460502 PMC4978862

[40] West JH, Hall PC, Arredondo V, Berrett B, Guerra B, Farrell J. Health behavior theories in diet apps. Journal of consumer health on the internet. 2013;17(1):10–24.

[41] Davis SF, Ellsworth MA, Payne HE, Hall SM, West JH, Nordhagen AL. Health Behavior Theory in Popular Calorie Counting Apps: A Content Analysis. JMIR mHealth and uHealth. 2016;4(1):e19. doi: 10.2196/mhealth.4177 26935898 PMC4795330

[42] Michie S, Johnston M. Theories and techniques of behaviour change: Developing a cumulative science of behaviour change. Taylor & Francis; 2012.

[43] Glanz K, Bishop DB. The role of behavioral science theory in development and implementation of public health interventions. Annual review of public health. 2010;31:399–418. doi: 10.1146/annurev.publhealth.012809.103604 20070207

[44] Wang Q, Egelandsdal B, Amdam GV, Almli VL, Oostindjer M. Diet and Physical Activity Apps: Perceived Effectiveness by App Users. JMIR mHealth uHealth. 2016;4(2):e33. doi: 10.2196/mhealth.5114 27056639 PMC4840256

[45] Braun V, Clarke V. Using thematic analysis in psychology. Qualitative research in psychology. 2006;3(2):77–101.

[46] Michie S, Richardson M, Johnston M, Abraham C, Francis J, Hardeman W, et al. The behavior change technique taxonomy (v1) of 93 hierarchically clustered techniques: building an international consensus for the reporting of behavior change interventions. Annals of behavioral medicine. 2013;46(1):81–95. doi: 10.1007/s12160-013-9486-6 23512568

[47] Tong A, Sainsbury P, Craig J. Consolidated criteria for reporting qualitative research (COREQ): a 32-item checklist for interviews and focus groups. International journal for quality in health care. 2007;19(6):349–57. doi: 10.1093/intqhc/mzm042 17872937

[48] Guest G, Bunce A, Johnson L. How many interviews are enough? An experiment with data saturation and variability. Field methods. 2006;18(1):59–82.

[49] Fusch PI, Ness LR. Are we there yet? Data saturation in qualitative research. The qualitative report. 2015;20(9):1408.

[50] Maher C, Hadfield M, Hutchings M, De Eyto A. Ensuring Rigor in Qualitative Data Analysis. International Journal of Qualitative Methods. 2018;17(1):160940691878636.

[51] Guba EG, Lincoln YS. Fourth generation evaluation: Sage; 1989.

[52] Connelly LM. Trustworthiness in qualitative research. Medsurg Nursing. 2016;25(6):435. 30304614

[53] Gottlieb BH, Bergen AE. Social support concepts and measures. Journal of psychosomatic research. 2010;69(5):511–20. doi: 10.1016/j.jpsychores.2009.10.001 20955871

[54] Chawla NV, Davis DA. Bringing big data to personalized healthcare: a patient-centered framework. Journal of general internal medicine. 2013;28(3):660–5. doi: 10.1007/s11606-013-2455-8 23797912 PMC3744281

[55] Stone D, Jarrett C, Woodroffe M, Minocha S. User interface design and evaluation: Elsevier; 2005.

[56] Hassenzahl M. User experience and experience design. The encyclopedia of human-computer interaction. 2013;2.

[57] Madeira RN, Germano H, Macedo P, Correia N. Personalising the user experience of a mobile health application towards Patient Engagement. Procedia Computer Science. 2018;141:428–33.

[58] Michie S, Abraham C, Whittington C, McAteer J, Gupta S. Effective techniques in healthy eating and physical activity interventions: a meta-regression. Health Psychology. 2009;28(6):690. doi: 10.1037/a0016136 19916637

[59] Wardle J, Parmenter K, Waller J. Nutrition knowledge and food intake. Appetite. 2000;34(3):269–75. doi: 10.1006/appe.1999.0311 10888290

[60] Health MMo. History of Food Composition Database: Malaysian Ministry of Health; 2015 [Available from: https://myfcd.moh.gov.my/index.php/about-us/history-of-food-composition-database.html.

