# Supplementary material for: Healthy adults’ views and experiences on behavior change strategies in mobile applications for diet monitoring: A single centre qualitative study
Source: PLoS One. 2023 Nov 16;18(11):e0292390. doi: 10.1371/journal.pone.0292390 (PMC10653402; doi:10.1371/journal.pone.0292390)
Supplement: S1 File — (DOCX) [file pone.0292390.s001.docx]

| **Code/ Categories** | **Subthemes** | **Themes** |
| --- | --- | --- |
| Identification of Achievement | Awareness of Progress | Instilling Self-Awareness |
| Tangible Rewards |  |  |
| App Feedback |  |  |
| Encouragement |  |  |
| Self Monitoring |  |  |
| Social Comparison |  |  |
| Outcome |  |  |
| Awareness of Calories | Awareness of Consequences |  |
| Awareness of Diet |  |  |
| Awareness of Satiety |  |  |
| Awareness of Weight Gain |  |  |
| Mood Tracking |  |  |
| Awareness of Repercussions |  |  |
| Awareness of Calories |  |  |
| Behaviour Tracking |  |  |
| Anticipated Regret |  |  |
| Mood Tracking |  |  |
| Social Media | In-App Community | Closed online group support |
| Media |  |  |
| Colleague / Work | Social Circle |  |
| Friends |  |  |
| Family |  |  |
| Advice From Health Professionals | Accurate and Reliable Information | Shaping Knowledge |
| Suggestions by App |  |  |
| Improved Knowledge on Diet | New Knowledge |  |
| Suggestions by App | Personalized Goal Setting | Personalization |
| Intermittent Fasting |  |  |
| Reminder to Perform Behaviour |  |  |
| Set Goals From Beginning |  |  |
| Automated Monitoring | Personal Tailoring of Features |  |
| Self-monitoring |  |  |
| Personalised Feedback |  |  |
| Customizable Features |  |  |
| Flexible Reminders |  |  |
| Sharing on Social Media | Privacy Concerns |  |
| Location Tracking |  |  |
| Automated Monitoring | Mental Stress |  |
| Self-monitoring |  |  |
| Restriction |  |  |
| Accessible | Ease of Use | User-Friendly Design |
| No Need For Tutorial |  |  |
| Easy to Find Required Food |  |  |
| Free Apps |  |  |
| Record Food At Any Time |  |  |
| Smooth Attractive Graphics | Smooth User Experience |  |
| Easy-to-Navigate Layout |  |  |
| Attainable Rewards |  |  |
