# Supplementary material for: Healthy adults’ views and experiences on behavior change strategies in mobile applications for diet monitoring: A single centre qualitative study
Source: PLoS One. 2023 Nov 16;18(11):e0292390. doi: 10.1371/journal.pone.0292390 (PMC10653402; doi:10.1371/journal.pone.0292390)
Supplement: S3 File — (PDF) [file pone.0292390.s003.pdf]

Version 7

MATLAMAT PENYELIDIKAN:	
• Apa motivasi, pengalaman, pendapat, dan kepatuhan para peserta untuk menggunakan aplikasi pencatatan pemakanan?(Wang et al 2016)<br>• Apakah sebab bagi orang dewasa yang tidak berniat menggunakan aplikasi pencatatan pemakanan?<br>• Apakah alasan orang dewasa yang pernah menggunakan aplikasi pelacakan diet pada masa lalu, tetapi memutuskan untuk menghentikan penggunaan aplikasi tersebut?	
**PENGENALAN**	
Memberi salam dan terima kasih<br>Perkenalkan diri<br>Setuju pada peraturan dan undang-undang (Kerahsiaan, pendapat orang lain, membungkam telefon bimbit, mengemukakan soalan)<br>Kebenaran: Rakaman audio<br>Nama Panggilan<br>Objektif FGD	
**PEMBUKAAN**	
a. Tanya soalan mudah yang berkenaan dengan tajuk<br>i. Apa khabar?<br>ii. Sudah berapa lama anda bekerja di…?<br>iii. Rasa sibuk atau tertekan? (Pastikan peserta tidak apa-apa, boleh mengambil sesi pendek ini sebagai sedikit masa lapang untuk berehat, sambil membincangkan sesuatu yang bermanfaat)<br>b. Adakah anda ingin membincangkan aplikasi penjejakan diet berasaskan internet dan strategi tingkah laku makan dengan lebih terperinci sepanjang temu ramah?	
**Seksyen 1:**	
**Domain**	**Soalan**
Latar Belakang / Pengalaman	1. Can you share whether you have had any experience using mobile phone for health-related matters?<br>Prompt: Which platform, website or applications are you using or have used?<br>Prompt: If participants listed a few, focus on mobile application use experience.

Environmental factors (Motivation and promoting factors)<br><br>Barriers and facilitators (person and environment interaction)	2. For those who have downloaded or used mobile health-related application, can you share with us the type of mobile application you use?<br><br>3. What motivated/triggered you to download the health-related application?<br>Prompt: Was it because you are trying to monitor your weight/lose weight/maintain current weight?<br><br>4. How long have you been using this application?<br><br>5. How frequently do you use the health application on your phone?<br>Prompt: a few times a day or a few times a week<br><br>6. How did you find out about the app, and how did you decide on which app to choose?<br>Prompt: Why did you choose this mobile application?<br>Prompt: Did you download the app on your own or did someone recommend it to you? (i.e. friend, family, doctor, trainer)<br><br>7. How do you learn to use this app?<br><br>8. Can you share the function of this mobile application?<br><br>9. What do you like about this app?<br>Prompt: What are the most useful features?<br>Prompt: What are your favorite features?<br><br>10. What you dislike about this app?<br>Prompt: What features in the app you use less?<br>Prompt: Any aspect of this app you found difficult?<br><br>11. What benefit you get by using this app?<br><br>12. What changes in your behavior (eating) you noticed while using this app?<br><br>13. Are there any other features in the app that facilitate your current/desired eating behavior? Any features you found to be useful?<br>Prompt: Can you please share what features you have found useful to support you in making changes in your eating behavior?<br>Prompt: What is your opinion on calories count features? Is this good to monitor your food intake?

	Prompt: How easy is the features to keep record on your calorie counting?<br>Prompt: What do you think about the food you had to give up?<br>Prompt: Do you think this app has changed the way you select your food?<br>Prompt: How easy to adopt to these changes?<br><br>14. Are there any other features in the app that hinder your current/desired eating behavior? Any features you found to be not useful?<br>15. Is there any activity you would not carry out if you did not have the health-related app?<br>Prompt: For example; counting calories, choosing your food, going to gym, counting footsteps<br><br>16. Do you talk about weight management with your friends, family, health care providers?<br>17. Are you using any support group to discuss your opinion, feelings and discuss about eating behavior?<br>18. What other supportive tool will assist in weight management?<br>19. If you could improve something about this app, to continue using it, what would it be? Why?<br>20. What would ensure you engage and continue using this app?<br>21. To non-users:<br>a) Is this technology sometimes you knew about?<br>b) Were you interested in any apps? Why?<br>c) What hindered from using any diet app?
**Section 2: Trigger Materials**	
Now I am going to show you some slides with features from health apps that are currently available in the market. Some of these features may have been brought up during the discussion earlier, but we are going to get deeper into them now, with some visual aids.<br><br>Here are some examples of general diet app interfaces:<br>Left to Right: MyNutriDiari2 by Nutrition Division, Ministry of Health Malaysia, MyNutriDiari2 by Nutrition Division, Ministry of Health Malaysia, Weight Loss Coach - Reduce Body Fat & Lose Weight by Droid Infinity	

Trigger 1: Goal setting
Some of the main features of diet & health apps currently are that they help you to set goals and plan. You can see here, these are aimed at weight management.<br><br>Left to right: MyNutriDiari2 by Nutrition Division, Ministry of Health Malaysia, MyNutriDiari2 by Nutrition Division, Ministry of Health Malaysia and DietCam by UMCH<br><br>1. What do you think of this feature? Do you like it?<br>2. Do you think it will motivate you to change your behaviour?<br>3. Any aspects of this feature that you have used before that you like?<br>4. Any aspect of this feature that you dislike? Any possible issues that you anticipate with this feature?
**Trigger 2: Advice, tips and health information**
Left to right: MyNutriDiari2 by Nutrition Division, Ministry of Health Malaysia, MyNutriDiari2 by Nutrition Division, Ministry of Health Malaysia, Calorie Counter - MyFitnessPal by MyFitnessPal, Inc<br><br>1. What are your thoughts on these features in the apps?<br>2. Are there any particular types of advice that would be useful for you? Why?<br>3. Is there any problem or concern you anticipate with this feature?<br>4. Any type of advice or tips from your experience with app use that you like or dislike?<br>5. Do you think a warning type of alert or advice is useful or desirable to you? Why?
**Trigger 3: Tools to monitor behaviour, mood and well being**
Behaviour: Left to right: DietCam by UMCH, Calorie Counter - MyFitnessPal by MyFitnessPal, Inc<br><br>Left to right: Calorie Counter - MyFitnessPal by MyFitnessPal, Inc, MyNutriDiari2 by Nutrition Division, Ministry of Health Malaysia, MyNutriDiari2 by Nutrition Division, Ministry of Health Malaysia<br><br>Camera: Calorie Counter - MyFitnessPal by MyFitnessPal, Inc, Calorie Counter - MyFitnessPal by MyFitnessPal, Inc, Calorie Mama AI: Meal Planner & Food Macro Counter by Azumio Inc.(Google Play Store Image)<br><br>All three images:Calorie Counter, Home Workout & Immunity Plan by HealthifyMe (Calorie Counter, Weight Loss Coach)Health & Fitness<br><br>Mood: All four images: Daylio - Diary, Journal Mood Tracker (Google Play Store Image)<br><br>1. What are your thoughts on these features?<br>2. Is there any problem or concern you anticipate with this feature?

3. Do you feel feedback from this feature is useful?<br>4. How do you usually monitor if without app? Why do you use that method?
**Trigger 4: Reminders and prompts**
Left to right, top to bottom (where applies):<br>Calorie Counter, Home Workout & Immunity Plan by HealthifyMe, Health Mate - Calorie counter & Weight Loss App by Pixel Bytes, Calorie Counter - MyFitnessPal by MyFitnessPal, Inc and Calorie Counter - MyFitnessPal by MyFitnessPal, Inc<br><br>1. What are your thoughts on these features? Do you like it?<br>2. Is there any problem or concern you anticipate with this feature?<br>3. Do you use any other reminders aside from app? What are they?<br>4. Are personalised reminders something useful? (sensing)
**Trigger 5: Sharing progress through social media**
In: Left to right: Calorie Counter - MyFitnessPal by MyFitnessPal,Calorie Counter - MyFitnessPal by MyFitnessPal and Runkeeper - GPS Track Run Walk by ASICS Digital, Inc.<br><br>Share: Left to right: MyNutriDiari2 by MoH Malaysia, PUMATRAC Home Workouts, Training, Running, Fitness by PUMA SE<br><br>1. What are your thoughts on these features? Do you like it?<br>2. Which type do you prefer? Why?<br>3. Which platforms do you use most often? Would you use the same platform if you were to share your progress? DO you share regardless?<br>4. Is there any problem or concern you anticipate with this feature?
**Trigger 6: Context sensing**
Left to right: Google Fit: Health & Activity Tracking by Google LLC, Google Fit: Health & Activity Tracking by Google LLC and AIA Vitality Weekly Challenge by AIA Technology Shared Services<br><br>1. What are your thoughts on these features?<br>2. What else would you like it to do?<br>3. Is there any problem or concern you anticipate with this feature?<br>4. Do you usually use the GPS or share location on your phone?
**Trigger 7: Entertainment Badges & Rewards**
Left to Right: Nike Training Club (app no longer available, image from Google https://www.pinterest.com/pin/334110866100853382/)<br>AIA Vitality<br>https://www.aiavitality.com.my/vitality_aia_my/web/linked_content/pdfs/rewards/Weekly_Chall

enge_Guide.pdf<br><br>1. What are your thoughts on these features?<br>2. What type of rewards would you be interested in?<br>3. How about simple audio reward? Non-monetary?<br>4. Is there any problem or concern you anticipate with this feature?
**CLOSING**
Is there anything else important that we have not already talked about that you think would help me understand this topic better?<br>Thank the participants.
