# Supplementary material for: Healthy adults’ views and experiences on behavior change strategies in mobile applications for diet monitoring: A single centre qualitative study
Source: PLoS One. 2023 Nov 16;18(11):e0292390. doi: 10.1371/journal.pone.0292390 (PMC10653402; doi:10.1371/journal.pone.0292390)
Supplement: S4 File — (PDF) [file pone.0292390.s004.pdf]

Integrating Behaviorial Strategies in a Mobile Diet App

Trigger Materials

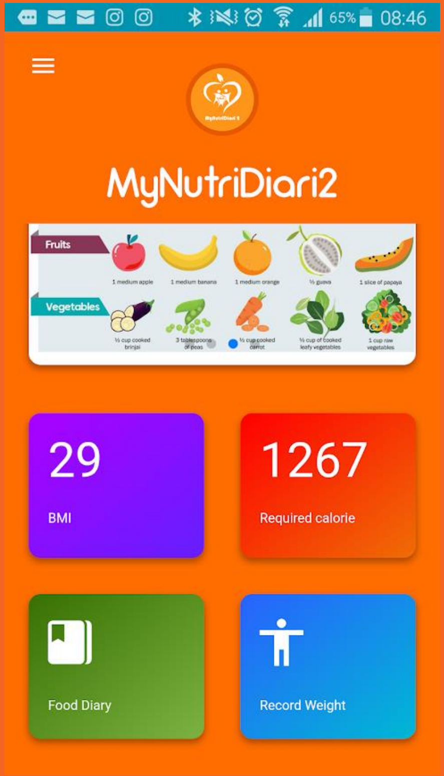
08:46
MyNutriDiari2
Fruits
Vegetables
29
BMI
1267
Required calorie
Food Diary
Record Weight

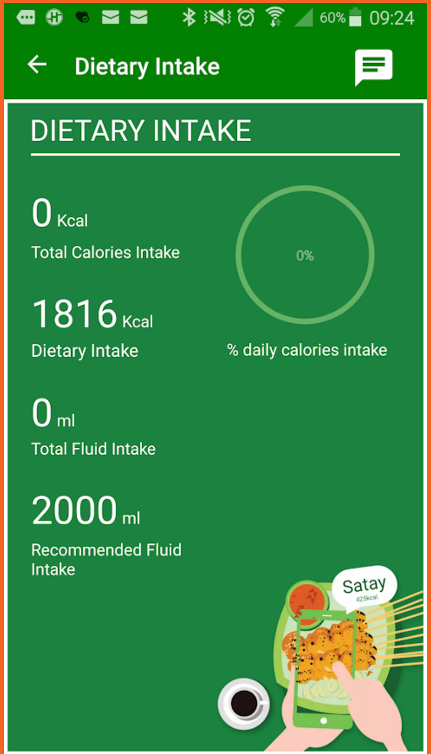
09:24
Dietary Intake
DIETARY INTAKE
0 Kcal
Total Calories Intake
0%
1816 Kcal
Dietary Intake
% daily calories intake
0 ml
Total Fluid Intake
2000 ml
Recommended Fluid Intake
Satay

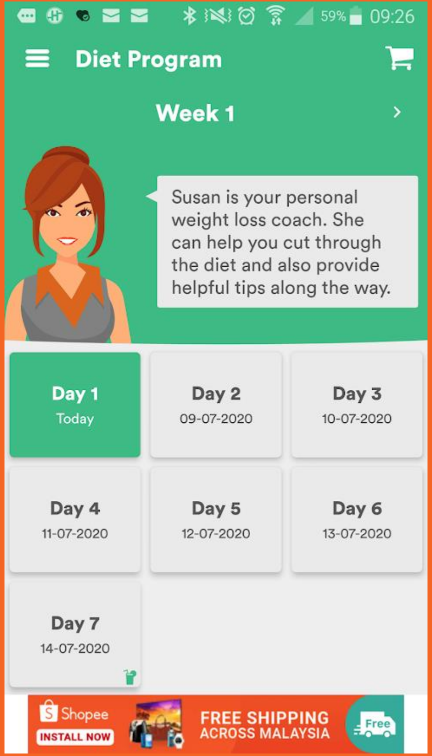
09:26
Diet Program
Week 1
Susan is your personal weight loss coach. She can help you cut through the diet and also provide helpful tips along the way.
Day 1
Today
Day 2
09-07-2020
Day 3
10-07-2020
Day 4
11-07-2020
Day 5
12-07-2020
Day 6
13-07-2020
Day 7
14-07-2020
Shopee
INSTALL NOW
FREE SHIPPING
ACROSS MALAYSIA
Free

Goal Setting

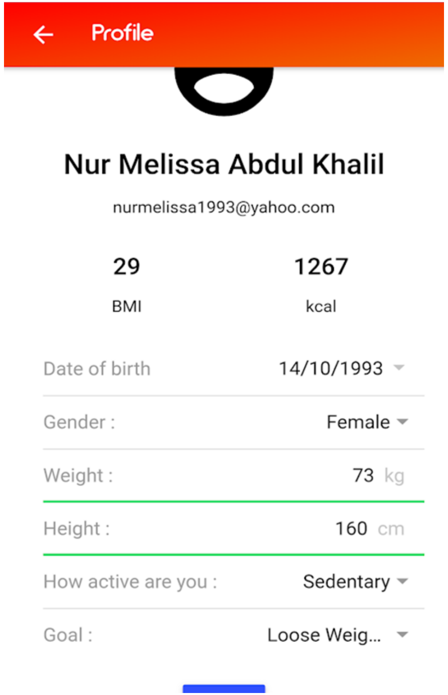

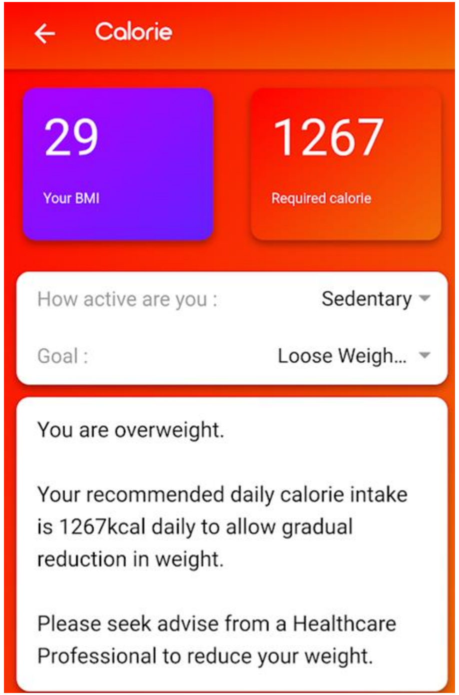

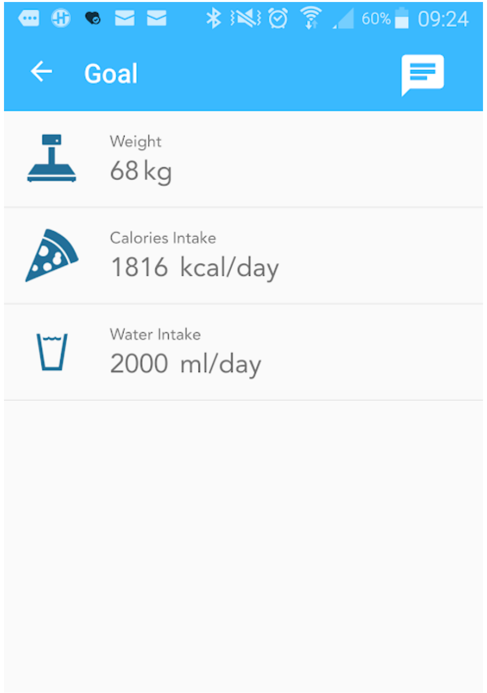

Advice, Tips & Health Information

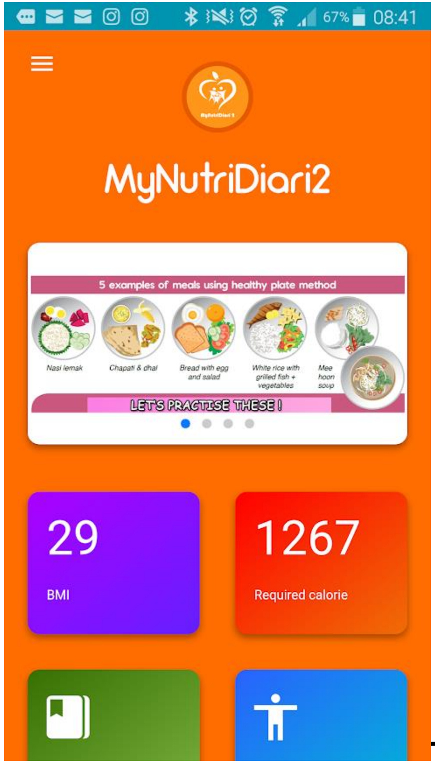

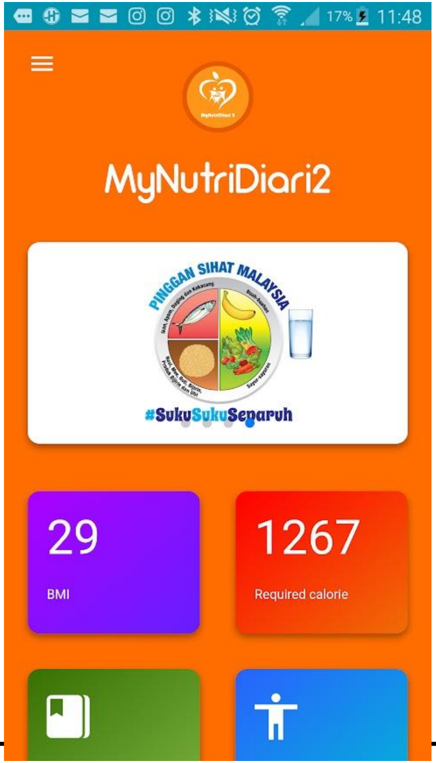

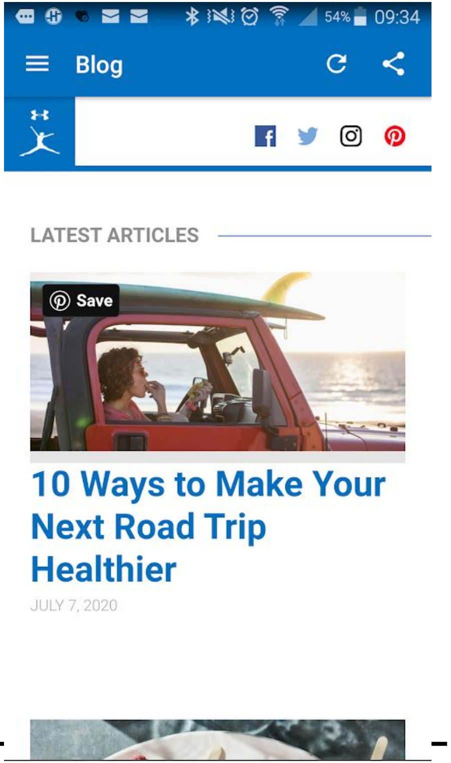

Tools to Monitor Behaviour, Mood & Well-being

Behaviour: Food Intake / Diary

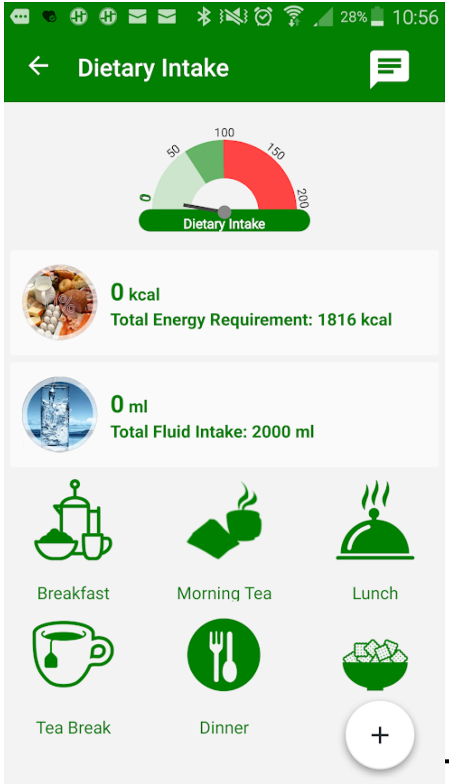

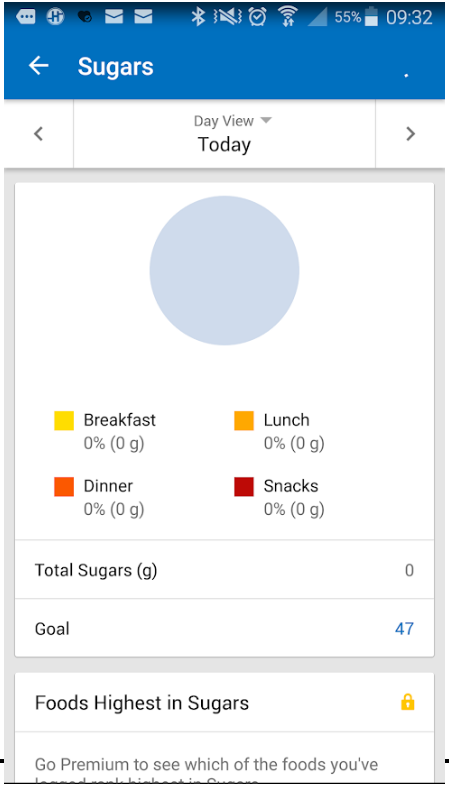

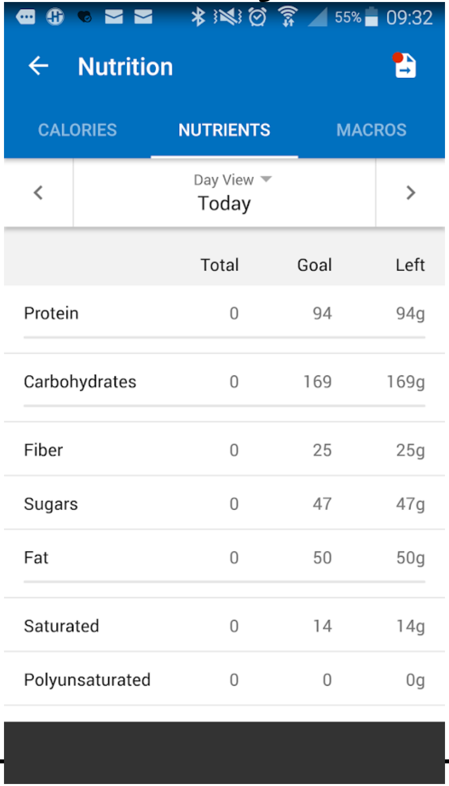

Behaviour: Food Intake / Diary

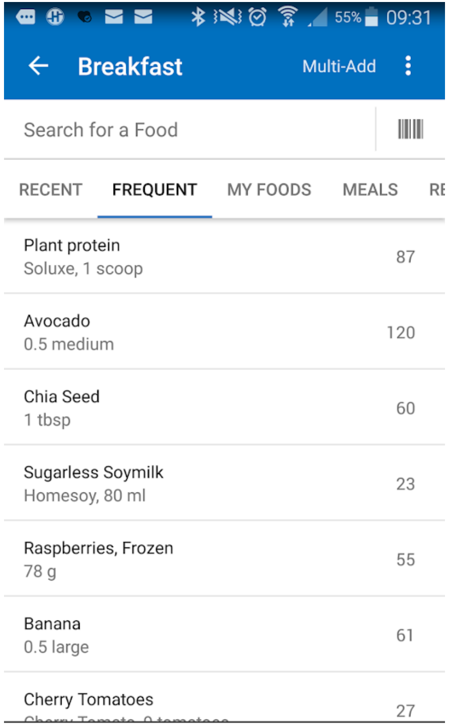

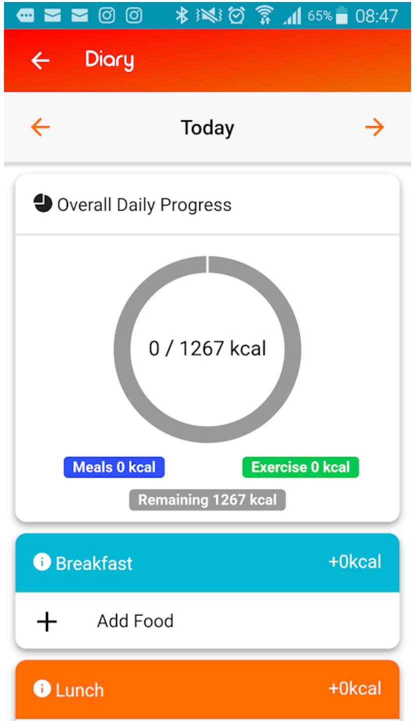

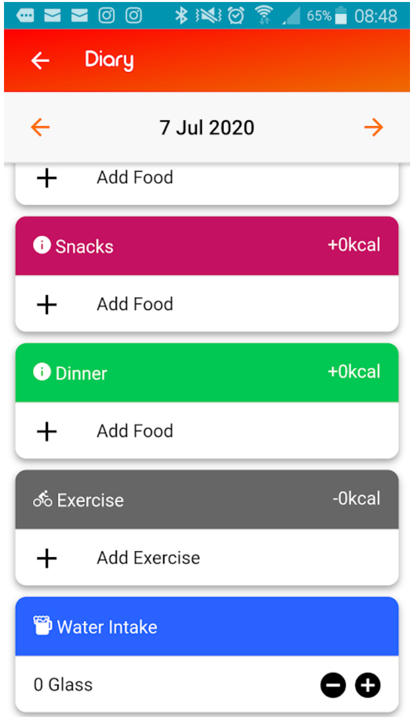

Camera

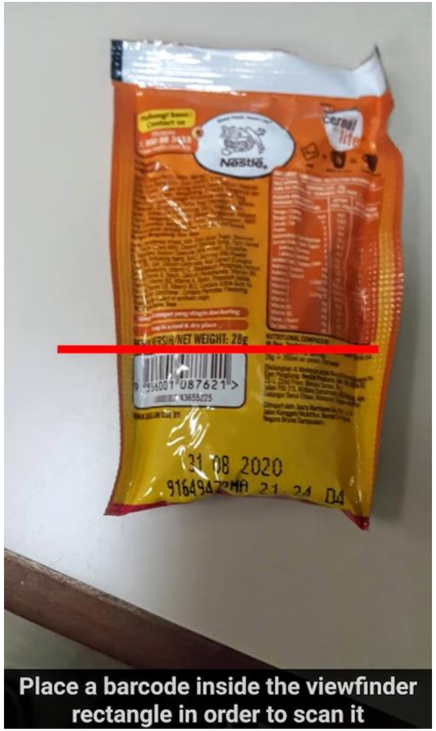
NET WEIGHT: 28g
31 08 2020
Place a barcode inside the viewfinder rectangle in order to scan it

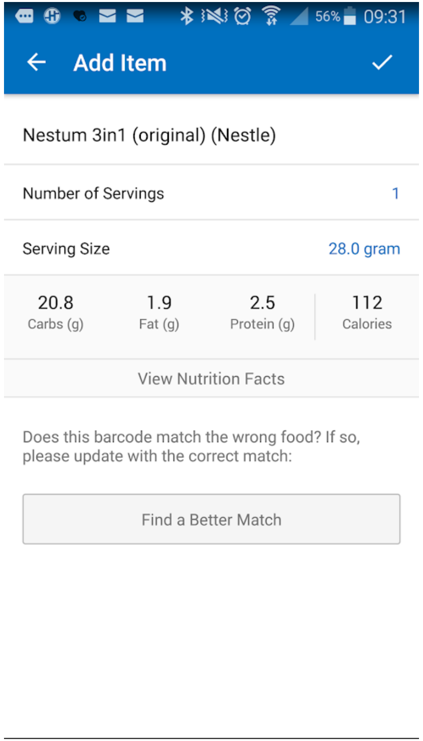
56% 09:31
Add Item
Nestum 3in1 (original) (Nestle)
Number of Servings 1
Serving Size 28.0 gram
20.8 Carbs (g)
1.9 Fat (g)
2.5 Protein (g)
112 Calories
View Nutrition Facts
Does this barcode match the wrong food? If so, please update with the correct match:
Find a Better Match

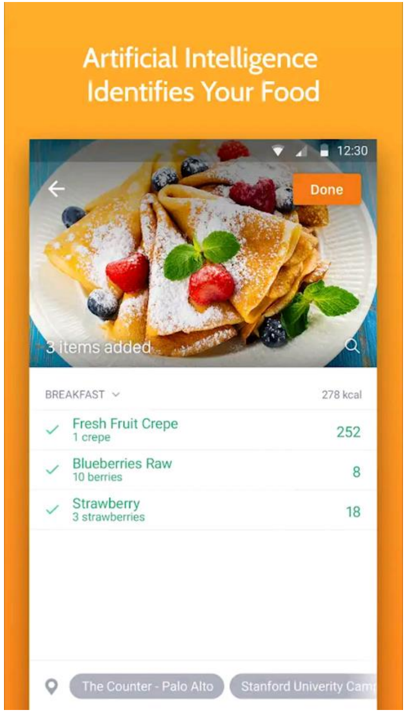
Artificial Intelligence Identifies Your Food
12:30
Done
3 items added
BREAKFAST
278 kcal
Fresh Fruit Crepe
1 crepe
252
Blueberries Raw
10 berries
8
Strawberry
3 strawberries
18
The Counter - Palo Alto

Camera

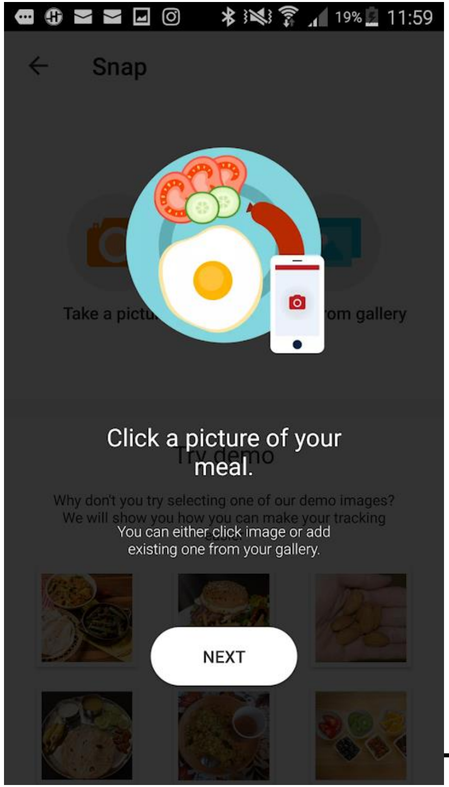
19% 11:59
Snap
Click a picture of your meal.
You can either click image or add existing one from your gallery.
NEXT

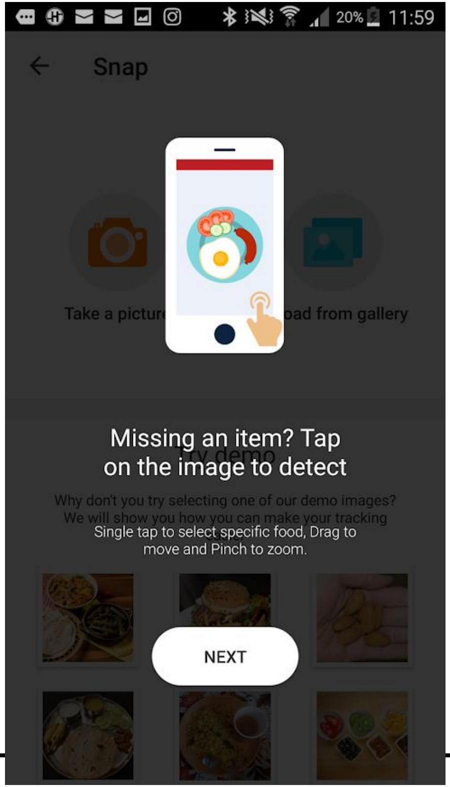
20% 11:59
Snap
Missing an item? Tap on the image to detect
Single tap to select specific food, Drag to move and Pinch to zoom.
NEXT

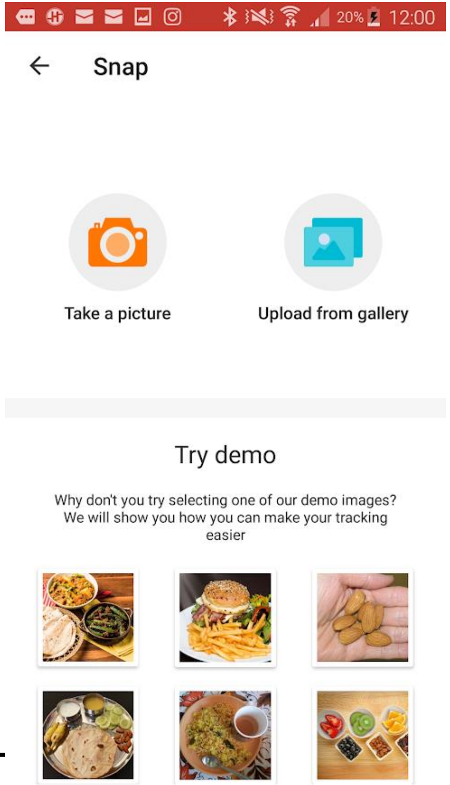
20% 12:00
Snap
Take a picture
Upload from gallery
Try demo
Why don't you try selecting one of our demo images? We will show you how you can make your tracking easier

Mood & Well-being

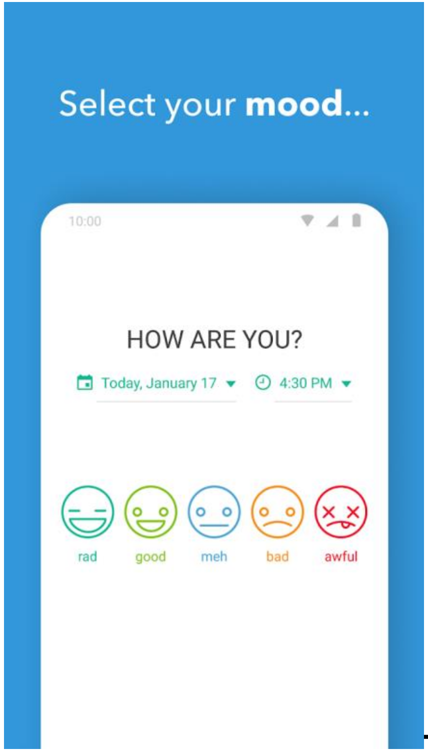

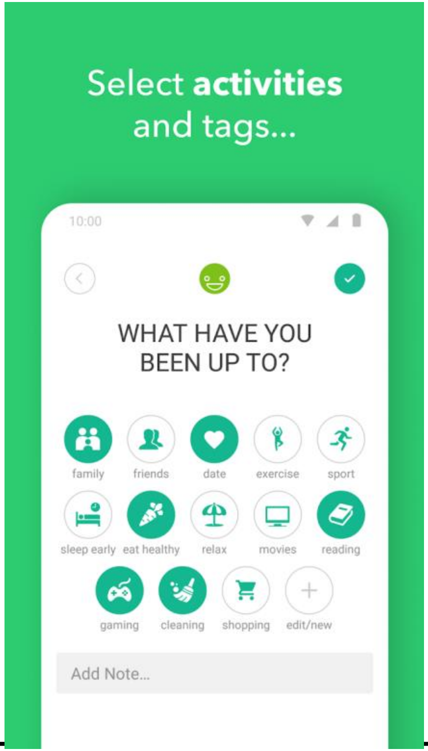

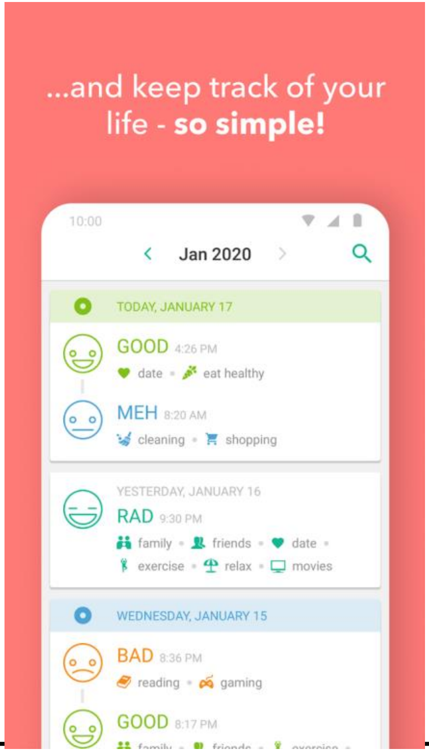

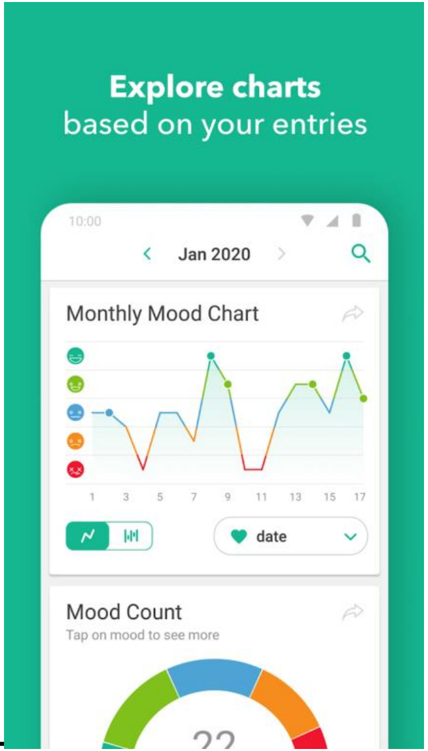

Reminders & Prompts

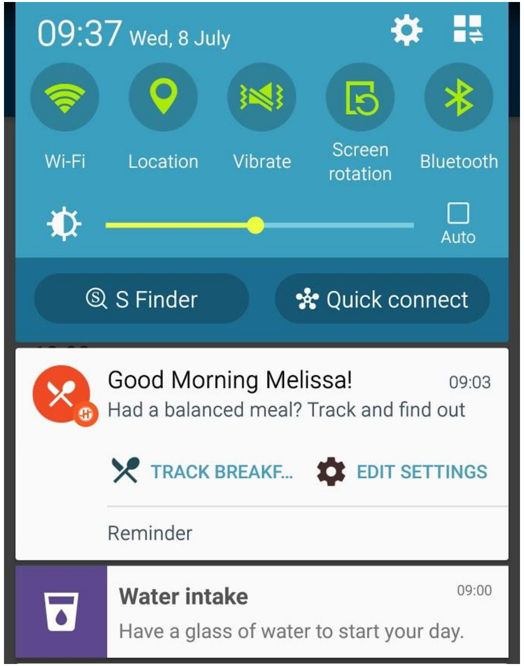

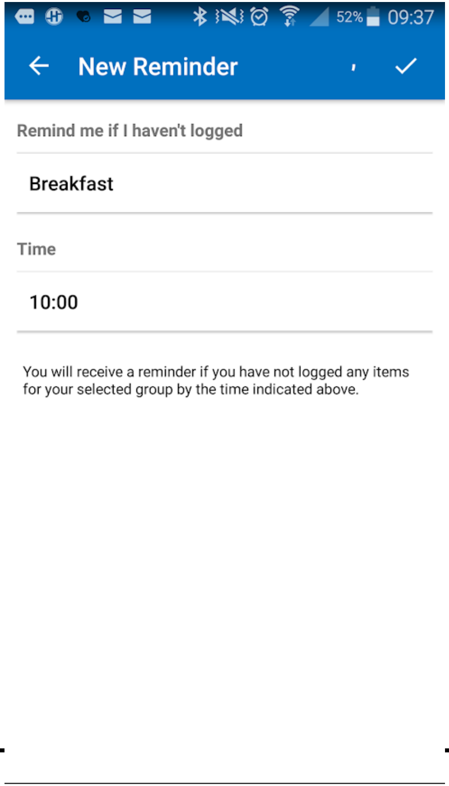

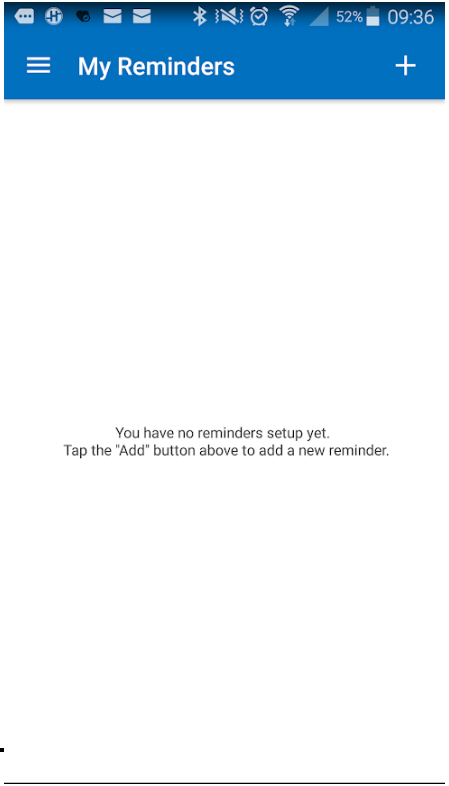

Sharing Your Progress Through Social Media

In- App Social Media

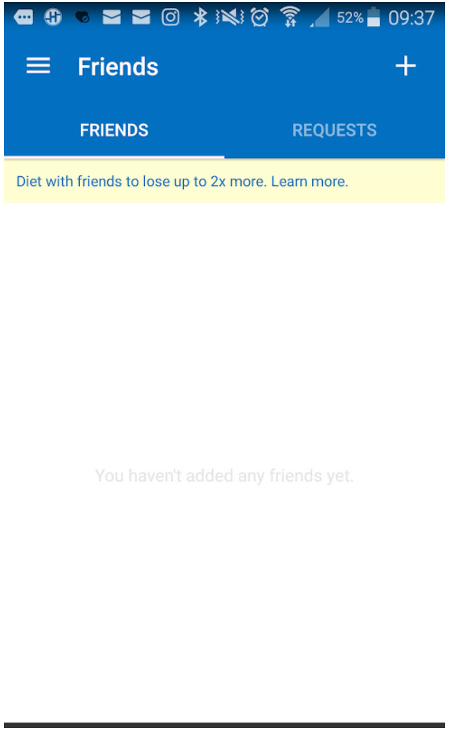

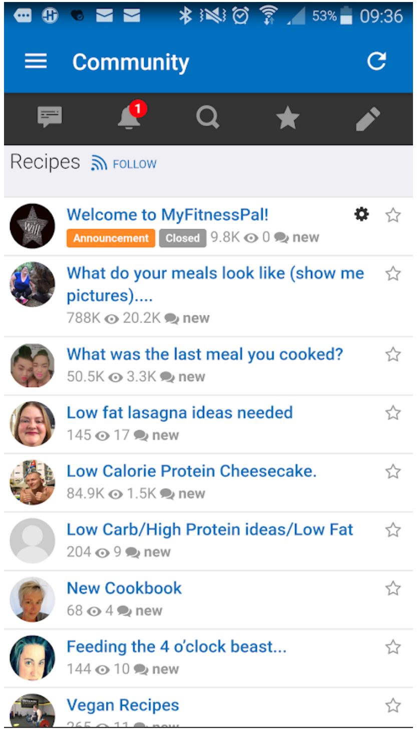

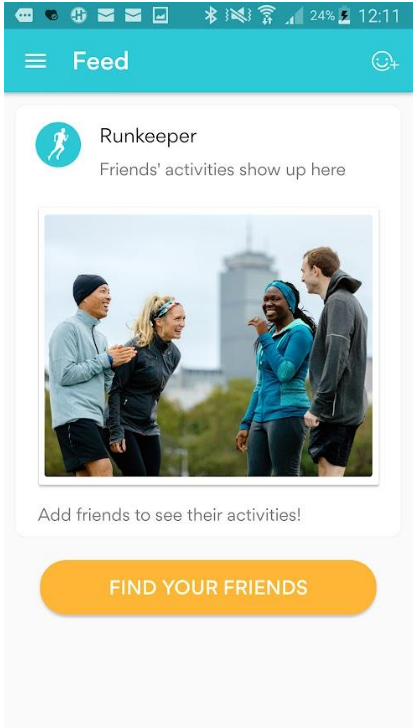

Share on Other Platforms

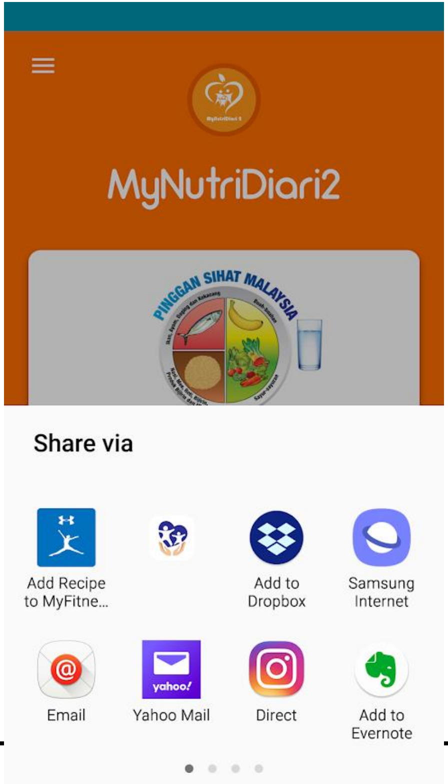

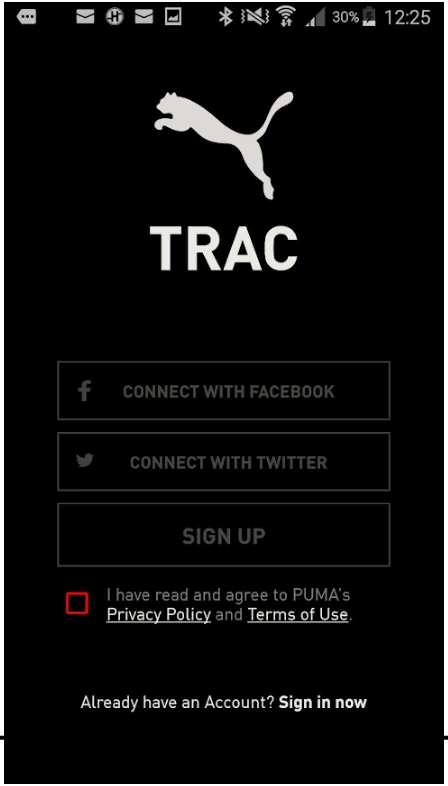

Context Sensing

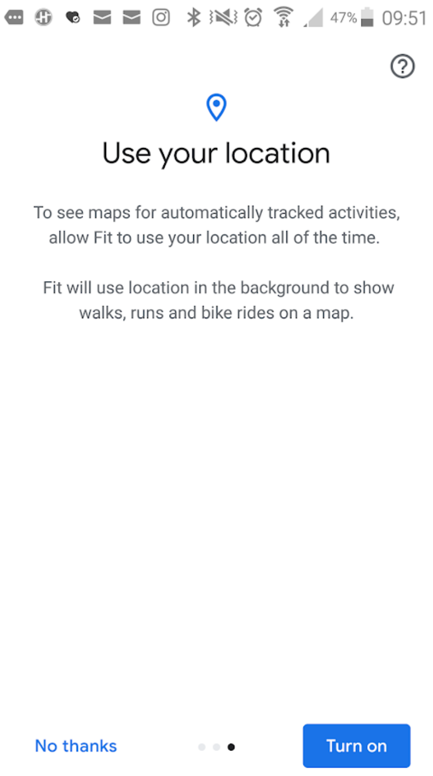

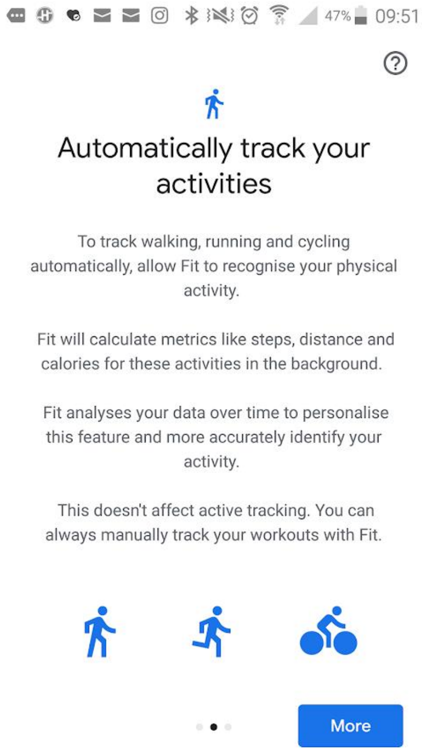

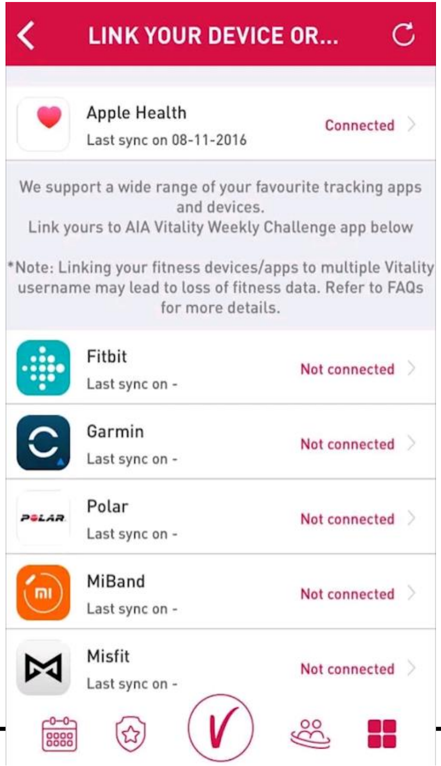

Entertainment Badges & Rewards

Badges

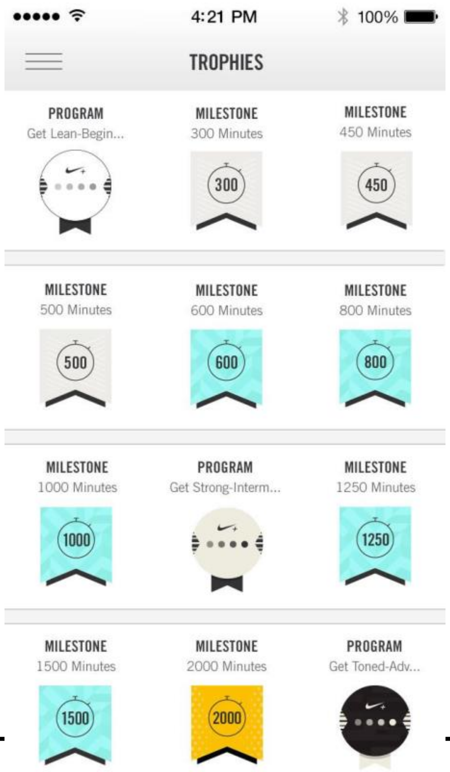
4:21 PM
100%
TROPHIES
PROGRAM
Get Lean-Begin...
MILESTONE
300 Minutes
300
MILESTONE
450 Minutes
450
MILESTONE
500 Minutes
500
MILESTONE
600 Minutes
600
MILESTONE
800 Minutes
800
MILESTONE
1000 Minutes
1000
PROGRAM
Get Strong-Interm...
MILESTONE
1250 Minutes
1250
MILESTONE
1500 Minutes
1500
MILESTONE
2000 Minutes
2000
PROGRAM
Get Toned-Adv...

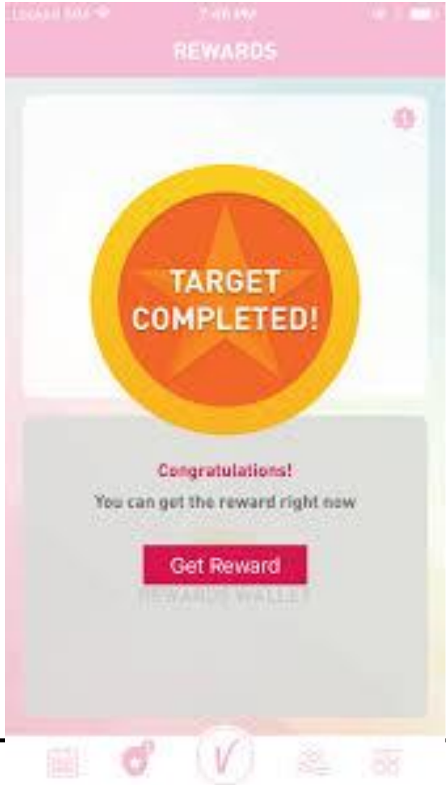
REWARDS
TARGET COMPLETED!
Congratulations!
You can get the reward right now
Get Reward

Badges

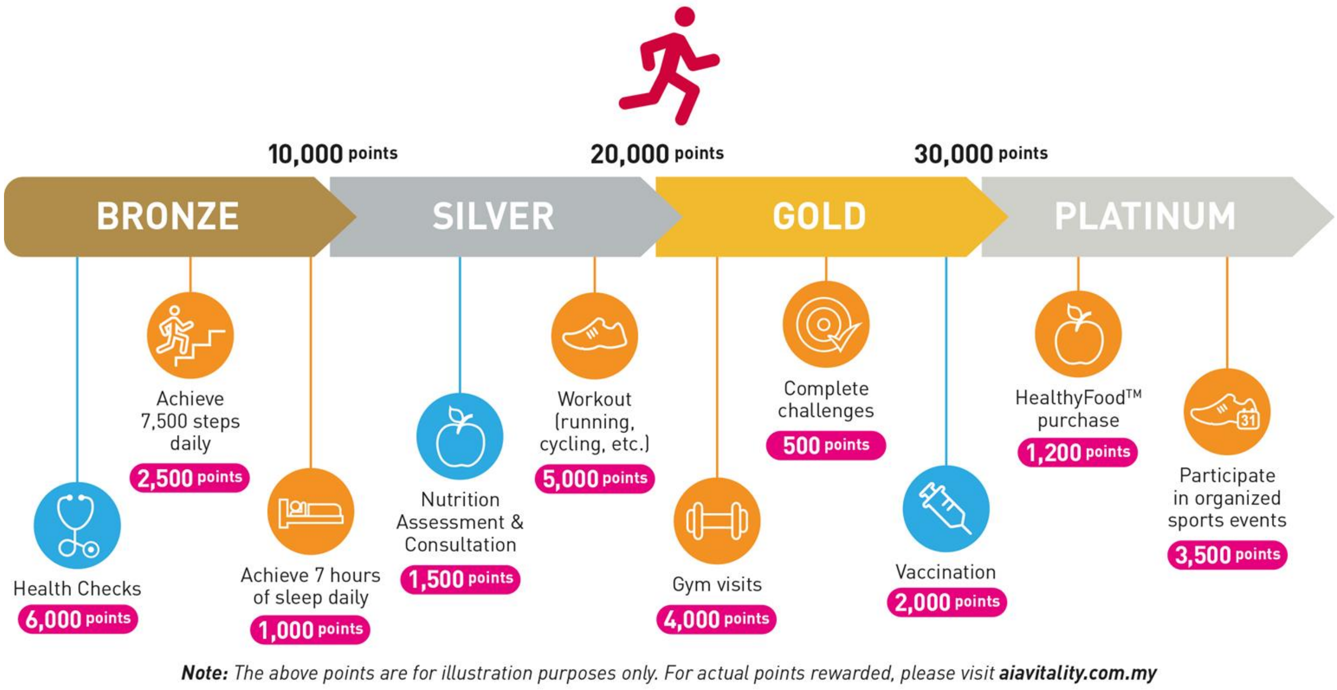
10,000 points
20,000 points
30,000 points
BRONZE
SILVER
GOLD
PLATINUM
Achieve 7,500 steps daily
2,500 points
Health Checks
6,000 points
Achieve 7 hours of sleep daily
1,000 points
Nutrition Assessment & Consultation
1,500 points
Workout (running, cycling, etc.)
5,000 points
Gym visits
4,000 points
Complete challenges
500 points
Vaccination
2,000 points
HealthyFood™ purchase
1,200 points
Participate in organized sports events
3,500 points
Note: The above points are for illustration purposes only. For actual points rewarded, please visit aiavitality.com.my

Rewards

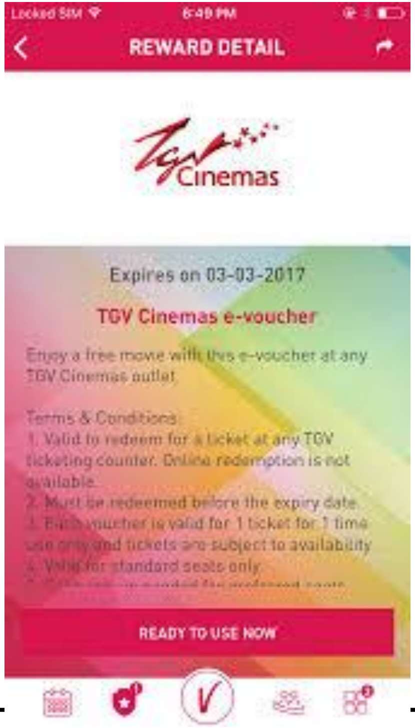
REWARD DETAIL
Cinemas
Expires on 03-03-2017
TGV Cinemas e-voucher
Enjoy a free movie with this e-voucher at any TGV Cinemas outlet
READY TO USE NOW

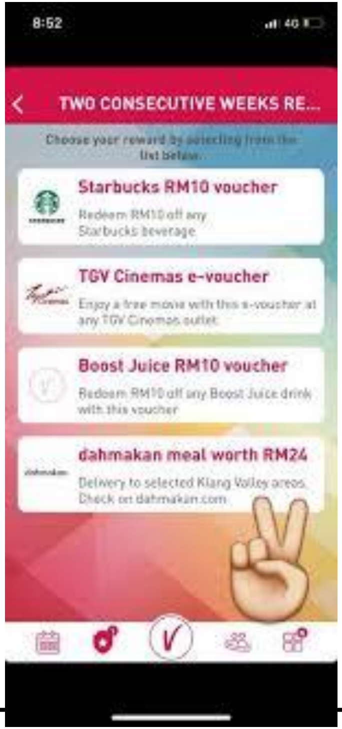
TWO CONSECUTIVE WEEKS RE...
Starbucks RM10 voucher
TGV Cinemas e-voucher
Boost Juice RM10 voucher
dahmakan meal worth RM24
